# Molecular Epidemiology and Clinical Characteristics of Drug-Resistant *Mycobacterium tuberculosis* in a Tuberculosis Referral Hospital in China

**DOI:** 10.1371/journal.pone.0110209

**Published:** 2014-10-10

**Authors:** Qi Wang, Susanna K. P. Lau, Fei Liu, Yanlin Zhao, Hong Min Li, Bing Xi Li, Yong Liang Hu, Patrick C. Y. Woo, Cui Hua Liu

**Affiliations:** 1 CAS Key Laboratory of Pathogenic Microbiology and Immunology, Institute of Microbiology, Chinese Academy of Sciences, Beijing, China; 2 State Key Laboratory of Emerging Infectious Diseases, Department of Microbiology, The University of Hong Kong, Hong Kong Special Administrative Region, China; 3 National Center for Tuberculosis Control and Prevention, Chinese Center for Disease Control and Prevention, Beijing, China; 4 Institute for Tuberculosis Research, the 309 Hospital, Beijing, China; Fudan University, China

## Abstract

**Background:**

Despite the large number of drug-resistant tuberculosis (TB) cases in China, few studies have comprehensively analyzed the drug resistance-associated gene mutations and genotypes in relation to the clinical characteristics of *M. tuberculosis* (Mtb) isolates.

**Methodology/Principal Findings:**

We thus analyzed the phenotypic and genotypic drug resistance profiles of 115 Mtb clinical isolates recovered from a tuberculosis referral hospital in Beijing, China. We also performed genotyping by 28 loci MIRU-VNTR analysis. Socio-demographic and clinical data were retrieved from medical records and analyzed. In total, 78 types of mutations (including 42 previously reported and 36 newly identified ones) were identified in 115 Mtb clinical isolates. There was significant correlation between phenotypic and genotypic drug resistance rates for first-line anti-TB drugs (*P*<0.001). Genotyping revealed 101 MIRU-VNTR types, with 20 isolates (17.4%) being clustered and 95 isolates (82.6%) having unique genotypes. Higher proportion of re-treatment cases was observed among patients with clustered isolates than those with unique MIRU-VNTR genotypes (75.0% vs. 41.1%). Moreover, clinical epidemiological links were identified among patients infected by Mtb strains belonging to the same clusters, suggesting a potential of transmission among patients.

**Conclusions/Significance:**

Our study provided information on novel potential drug resistance-associated mutations in Mtb. In addition, the genotyping data from our study suggested that enforcement of the implementation of genotyping in diagnostic routines would provide important information for better monitor and control of TB transmission.

## Introduction

Tuberculosis (TB) remains a major infectious and deadly disease in the world. In addition, multidrug resistance (MDR) and extensively drug-resistance (XDR) pose a more serious problem for TB control. WHO reported that about 8.6 million people developed TB, and 1.3 million died from the disease in 2012. India and China alone accounted for 26% and 12% of global cases, respectively. In addition, the global estimate of the burden of MDR-TB was 300, 000 cases among notified TB patients in 2012. India and China were the two countries estimated to have the largest numbers of TB patients with MDR-TB [1]. Therefore, there is an urgent need to better understand the molecular epidemiology and clinical characteristics of drug-resistant *M. tuberculosis* (Mtb) isolates, so as to provide knowledge for rapid molecular diagnosis for and better management of drug-resistant TB.

Despite the high TB burden and large number of drug-resistant TB cases in China, relatively few studies have performed comprehensive analysis of drug resistance-associated mutations of clinical Mtb isolates, their genotypes, as well as their association with clinical characteristics. We previously observed high rates of MDR- and XDR-TB among patients from a TB referral hospital in Beijing, China [2]. In addition, we established a TBDReaMDB database-coupled automatic analysis for drug resistance-associated mutations in Mtb isolates [3]. In this study, we further applied this method to perform comprehensive analysis of the drug resistance-associated mutations (genotypic drug resistance profiles) for a larger sample of Mtb clinical isolates from hospitalized TB patients. Molecular genotyping is a useful tool for analyzing Mtb strain diversity and transmission patterns, especially in high incidence settings such as TB referral hospitals. Genotyping methods based on variable number of tandem repeats (VNTR) of mycobacterial interspersed repetitive units (MIRU) have become popular methods used for TB epidemiological analysis [4], [5]. We thus also evaluated the genotypic population structure for the Mtb isolates based on 28 loci MIRU-VNTR genotyping analysis and its correlation with drug resistance associated mutations and clinical characteristics and epidemiology. Our study added to the growing list of potential TB drug resistance-associated mutations, which could be invaluable in applying MDR- and XDR-TB molecular detection tests. In addition, the genotyping data from our study suggested that enforcement of the implementation of genotyping in diagnostic routines would provide important information for better monitor and control of TB transmission.

## Materials and Methods

### Ethics statement

The investigation protocols used in this study were approved by the institutional ethics committee of the 309 Hospital, Beijing, China. Written informed consent for Mtb isolates to be collected as well as for their information to be stored in the hospital database for research purposes was provided by participants. Written informed consent was obtained from the next of kin, caretakers, or guardians on the behalf of the minors/children participants involved in this study. Permission for using the information in the medical records of the patients for research purposes was obtained from the 309 Hospital. The Institute ethics committee of the 309 Hospital and the Institute of Microbiology, Chinese Academy of Sciences approved this study.

### Mtb isolates, drug susceptibility testing and detection of drug resistance-associated mutations

Mtb isolates used in this study were obtained from TB patients being treated in the 309 Hospital, a TB referral hospital located in Beijing, China over a 3-year period (January 1, 2009–December 31, 2011). A total of 3860 culture positive TB patients were diagnosed and treated in the 309 Hospital during 2009–2011. Of these, 150 non-repetitive Mtb isolates were randomly selected to obtain pure cultures for further confirmation by p-nitrobenzoic acid and thiophene carboxylic acid hydrazine resistance tests as well as 16S rDNA sequencing analysis. Thirty-five isolates were excluded as a result of contaminated cultures or ambiguous sequencing results. The remaining 115 isolates were then subjected to DST, sequencing for drug resistance-associated gene mutations, and genotyping analysis. Epidemiologic and clinical data of the patients were extracted from the subjects’ medical records. Mtb isolates were cultured using the BACTEC 960 system (Becton Dickinson Diagnostic Systems, Sparks, MD, USA) according to the manufacturer’s instructions. The drug susceptibility testing (DST) were conducted according to the WHO guidelines as described previously [2]. Identification of drug resistance-associated mutations of Mtb isolates was conducted as described previously [3]. Written informed consent was obtained from participants.

### MIRU-VNTR genotyping of Mtb isolates

Deletion-targeted multiplex PCR (DTM-PCR) method was used initially to identify the Beijing strains [6]. The Mtb isolates were further genotyped by a 28 loci MIRU-VNTR method based on the 24 loci described by Supply et al. [4], [7] as well as the four hyper variable loci (1982, 3232, 3820, and 4120) described by Gao et al. [8] and Supply et al. [9]. The amplicons were evaluated on the 2% standard agarose gels by using a 100-bp DNA ladder (Takara), and sizing of the various VNTR alleles was done with the Peak Scanner Software v1.0 (PE Applied Biosystems). The number of repeats at each locus of MIRUs was determined and numerical values were assigned accordingly. Genetic clusters were defined as a group of two or more isolates exhibiting the same MIRU-VNTR pattern. The reference strain H37Rv was run as an additional control.

### Phylogenetic analysis

The phylogenetic tree based on the 28 loci MIRU-VNTR data was constructed using PAUP 4.0b software [10] with UPGMA (unweighted pair-group method with arithmetic means) method.

### Statistical analyses

All data were analyzed using the SPSS software (15.0 version). Comparisons of categorical variables were performed using the Pearson Chi-square test to compare different groups. A *P* value of <0.05 was considered to be statistically significant.

## Results

### General characteristics of the study subjects

A total of 115 non-repetitive Mtb isolates collected during 2009–2011 period were included in this study. The TB patients from whom the Mtb isolates were obtained included 53 susceptible TB cases, 21 MDR-TB cases, 17 XDR-TB cases, and 24 other types of TB cases (drug-resistant TB cases excluding MDR- and XDR-TB). All cases were HIV negative. The median age was 37 (range 14–86). Sixty-nine (60.0%) patients were male and 46 (40.0%) were female. Sixty-one (53.0%) patients were new cases and 54 (47.0%) were re-treatment cases. More detiled information on socio-demographic and clinical characteristics of the patients is shown in Table S1.

### Drug resistance-associated gene mutations in Mtb isolates

We identified 78 types of mutations in drug resistance-associated genes, including 42 previously reported ones (Table 1) and 36 newly identified ones (Table 2 and Table 3). More detailed information on the PCR primers used for as well as the mutations identified in drug resistance-associated loci in all Mtb isolates were listed in Table S2 and Table S3, respectively. There was significant correlation between phenotypic and genotypic drug resistance rates for first-line anti-TB drugs (*P<*0.001 for isoniazid, rifampicin, streptomycin, ethambutol and pyrazinamide by chi-square test). The percentages of genotypic resistant isolates among phenotypic resistant isolates for first-line anti-TB drugs varied between 40.0% to 95.7%. There was also significant correlation between some of the second-line anti-TB drugs (*P*<0.001 for ofloxacin/levofloxacin and kanamycin by chi-square test). The percentages of genotypic resistant isolates among phenotypic resistant isolates for second-line anti-TB drugs varied between 0% to 41.5% (Table S4).

**Table 1:** Previously reported mutations identified in drug resistance-associated loci in *M. tuberculosis* isolates.

Genes	BaseMutations	Aminoacidmutations	Drugs^a^	No. ofgenotypicresistantisolates/No. ofphenotypicresistantisolates	No. ofgenotypicresistantisolates/No. ofphenotypicsusceptibleisolates	*P* value	Mutation categories
Rv0341 (*iniB*)	CTGGTGTCGGCG665 (del)	-	INH	3/47	0/68	0.035	Drug resistance mutation
Rv1483 (*mabA*)	C-15T	-	INH	1/47	0/68	0.227	Phylogenetic informative mutation
Rv1483 (*mabA*)	T-8C	-	INH	3/47	0/68	0.035	Drug resistance mutation
Rv1908c (*katG*)	G944C	S315T	INH	14/47	0/68	<0.001	Drug resistance mutation
Rv1908c (*katG*)	G1389T	R463L	INH	43/47	0/68	<0.001	Drug resistance mutation
Rv2247 (*accD6*)	A686G	D229G	INH	43/47	0/68	<0.001	Drug resistance mutation
Rv2428 (*ahpC*)	G-51A	-	INH	1/47	0/68	0.227	Phylogenetic informative mutation
Rv2428 (*ahpC*)	C-52T	-	INH	1/47	0/68	0.227	Phylogenetic informative mutation
Rv2428 (*ahpC*)	G-48A	-	INH	1/47	0/68	0.227	Phylogenetic informative mutation
Rv0667 (*rpoB*)	T1289C	L511P	RMP	2/39	0/76	0.046	Drug resistance mutation
Rv0667 (*rpoB*)	A1291G	S512G	RMP	1/39	0/76	0.161	Phylogenetic informative mutation
Rv0667 (*rpoB*)	A1304G	D516G	RMP	1/39	0/76	0.161	Phylogenetic informative mutation
Rv0667 (*rpoB*)	A1304T	D516V	RMP	1/39	0/76	0.161	Drug resistance mutation^b^
Rv0667 (*rpoB*)	A1304C	D516A	RMP	1/39	0/76	0.161	Phylogenetic informative mutation
Rv0667 (*rpoB*)	C1333G	H526D	RMP	3/39	0/76	0.046	Drug resistance mutation
Rv0667 (*rpoB*)	A1334G	H526R	RMP	1/39	0/76	0.161	Phylogenetic informative mutation
Rv0667 (*rpoB*)	C1349T	S531L	RMP	13/39	0/76	<0.001	Drug resistance mutation
Rv0682 (*rpsL*)	A128G	K43R	SM	13/37	0/78	<0.001	Drug resistance mutation
Rv0682 (*rpsL*)	A263G	K88R	SM	3/37	0/78	0.011	Drug resistance mutation
Rv3919c (*gidB)*	C413T	A138V	SM	1/37	0/78	0.145	Phylogenetic informative mutation
Rv3919c (*gidB)*	A276C	E92D	SM	37/37	59/78	0.001	Phylogenetic informative mutation^c^
Rv3794 (*embA*)	C-12T	-	EMB	2/34	0/81	0.028	Drug resistance mutation
Rv3794 (*embA*)	G1049A	G350D	EMB	1/34	0/81	0.121	Phylogenetic informative mutation
Rv3795 (*embB*)	A916G	M306V	EMB	6/34	0/81	<0.001	Drug resistance mutation
Rv3795 (embB)	G918A	M306I	EMB	1/34	0/81	0.121	Phylogenetic informative mutation
Rv3795 (*embB*)	G982T	D328Y	EMB	1/34	0/81	0.121	Phylogenetic informative mutation
Rv3795 (*embB*)	G1217C	G406A	EMB	3/34	2/81	0.127	Phylogenetic informative mutation
Rv3795 (*embB*)	G1216A	G406S	EMB	1/34	0/81	0.121	Phylogenetic informative mutation
Rv3795 (*embB*)	G1217A	G406D	EMB	1/34	0/81	0.121	Phylogenetic informative mutation
Rv3795 (*embB*)	G1412C	R471P	EMB	0/34	1/81	0.515	Phylogenetic informative mutation
Rv3795 (*embB*)	C1489A	Q497K	EMB	1/34	0/81	0.121	Phylogenetic informative mutation
Rv3795 (*embB*)	A1490G	Q497R	EMB	0/34	1/81	0.515	Phylogenetic informative mutation
Rv2043c (*pncA*)	T384G	V128G	PZA	3/15	0/100	<0.001	Drug resistance mutation
Rv2043c (*pncA*)	GG391 (ins)	-	PZA	2/15	0/100	<0.001	Drug resistance mutation
Rv2043c (*pncA*)	C227T	T76I	PZA	1/15	0/100	0.010	Drug resistance mutation
Rv0006 (*gyrA*)	A281G	D94G	OFX,LVX	10/41	0/74	<0.001	Drug resistance mutation
Rv0006 (*gyrA*)	G284C	S95T	OFX,LVX	41/41	67/74	0.042	Phylogenetic informative mutation^d^
Rv0006 (*gyrA*)	C269T	A90V	OFX,LVX	5/41	0/74	0.002	Drug resistance mutation
Rv0006 (*gyrA*)	G280A	D94N	OFX,LVX	1/41	0/74	0.177	Phylogenetic informative mutation
Rv0006 (*gyrA*)	G280T	D94Y	OFX,LVX	1/41	0/74	0.177	Phylogenetic informative mutation
Rvnr01 (rrs)	G1332A	-	KAN	2/20	1/95	0.023	Drug resistance mutation
Rvnr01 (*rrs*)	A1401G	-	KAN	2/20	0/95	0.002	Drug resistance mutation

a INH, isoniazid; RMP, rifampicin; SM, streptomycin; EMB, ethambutol; PZA, pyrazinamide; OFX, ofloxacin; LVX, levofloxacin; KAN, kanamycin.

b Previously known to be associated with drug resistance (Luo T et al., Antimicrobial Agents and Chemotherapy, 2010).

c Previously known not associated with drug resistance (Spies FS et al., Journal of Clinical Microbiology, 2011).

d Previously known not associated with drug resistance (Giannoni F et al., Antimicrobial Agents and Chemotherapy, 2005).

**Table 2:** Newly identified mutations which happened alone in drug resistance-associated loci in *M. tuberculosis* isolates.

Genes	Basemutations	Amino acidmutations	Drugs^a^	No. of genotypicresistantisolates/No. ofphenotypicresistant isolates	No. ofGenotypicresistantisolates/No. ofphenotypicsusceptibleisolates	*P* value	Mutation categories
Rv0667 (*rpoB*)	A1198G	T481A	RMP	1/39	0/76	0.161	Phylogenetic informativemutation
Rv0667 (*rpoB*)	A1081(del)		RMP	3/39	0/76	0.014	Potential drug-resistantmutation
Rvnr01 (rrs)	A908C	-	SM	3/37	0/78	0.011	Potential drug-resistantmutation
Rv3919c (*gidB*)	C356A	A119D	SM	4/37	0/78	0.003	Potential drug resistancemutation
Rv3919c (*gidB*)	G579C	A193	SM	4/37	0/78	0.003	Potential drugresistance mutation
Rv3795 (*embB*)	C584T	P195L	EMB	0/34	1/81	0.515	Phylogeneticinformative mutation
Rv3795 (*embB*)	G940C	G314R	EMB	1/34	0/81	0.121	Phylogeneticinformative mutation
Rv3795 (*embB*)	A386G	N129S	EMB	1/34	0/81	0.121	Phylogenetic informative mutation
Rv3795 (*embB*)	A1602T	D534	EMB	1/34	0/81	0.121	Phylogenetic informativemutation
Rv3795 (*embB*)	A1687C	I563L	EMB	1/34	0/81	0.121	Phylogeneticinformative mutation
Rv3795 (*embB*)	G2247(del)		EMB	1/34	0/81	0.121	Phylogeneticinformative mutation
Rv3795 (*embB*)	G2067A	A609	EMB	1/34	0/81	0.121	Phylogeneticinformative mutation
Rv2043c (*pncA*)	A28(del)		PZA	1/15	0/100	0.010	Potential drug resistance mutation
Rv2043c (*pncA*)	G232T	G78C	PZA	0/15	1/100	0.697	Phylogeneticinformative mutation

a INH, isoniazid; RMP, rifampicin; SM, streptomycin; EMB, ethambutol; PZA, pyrazinamide; OFX, ofloxacin; LVX, levofloxacin; KAN, kanamycin; ETH, ethionamide.

**Table 3:** Newly identified mutations which happened together with other known resistance mutations in drug resistance-associated loci in *M. tuberculosis* isolates.

Genes	Base mutations	Amino acidmutations	Drugs^a^	No. ofGenotypicresistantisolates/No. ofphenotypicresistant isolates	No. ofgenotypicresistantisolates/No. ofphenotypicsusceptibleisolates	*P* value	Mutation categories
Rv0340	G444A	G148	INH	1/47	0/68	0.227	Phylogeneticinformative mutation
Rv1592c	T70 (del)	-	INH	47/47	68/68	No data	Referencespecific mutation
Rv1908c (*katG*)	T511G	D171A	INH	1/47	0/68	0.227	Phylogenetic informativemutation
Rv1908c (*katG*)	A566C	C189G	INH	1/47	0/68	0.227	Phylogeneticinformative mutation
Rv2247 (*accD6*)	G747C	T249	INH	1/47	0/68	0.227	Phylogenetic informativemutation
Rv2247 (*accD6*)	T759C	D253	INH	1/47	0/68	0.227	Phylogeneticinformative mutation
Rv2247 (*accD6*)	G662C	S221T	INH	1/47	0/68	0.227	Phylogenetic informativemutation
Rv2247 (*accD6*)	CG761AC	A254D	INH	1/47	0/68	0.227	Phylogeneticinformative mutation
Rv2428 (*ahpC*)	G119A	S40N	INH	1/47	0/68	0.227	Phylogeneticinformative mutation
Rv2428 (*ahpC*)	C-81T	-	INH	1/47	0/68	0.227	Phylogeneticinformative mutation
Rv2846c (*efpA*)	T368 (del)	-	INH	1/47	0/68	0.227	Phylogenetic informative mutation
Rv0667 (rpoB)	C1819A	R688S	RMP	1/39	0/76	0.161	Phylogenetic informative mutation
Rv0667 (*rpoB*)	A1789(del)		RMP	4/39	0/76	0.004	Potential drug resistant mutation
Rv3919c (*gidB*)	T615C	A205	SM	21/37	59/78	0.012	Potential drugresistant mutation?
Rv3919c (*gidB*)	G299A	S100F	SM	37/37	78/78	No data	Reference specific mutation
Rv3793 (*embC*)	C2379T	D793	EMB	0/34	1/79	0.515	Phylogenetic informative mutation
Rv3793 (*embC*)	C2781T	R927	EMB	34/34	81/81	No data	Referencespecific mutation
Rv3794 (*embA*)	C228T	C76	EMB	34/34	79/81	0.355	Phylogenetic informative mutation
Rv3795 (*embB*)	G524C	G175A	EMB	1/34	0/81	0.121	Phylogenetic informative mutation
Rv0006 (*gyrA*)	G61C	E21Q	OFX, LVX	41/41	68/74	0.061	Phylogenetic informative mutation
Rv1694 (*tlyA*)	A33G	L11	KAN	20/20	93/95	0.513	Phylogenetic informative mutation
Rv3854c (*ethA*)	A1080C	Q360H	ETH	17/17	98/98	No data	Reference specific mutation

a INH, isoniazid; RMP, rifampicin; SM, streptomycin; EMB, ethambutol; PZA, pyrazinamide; OFX, ofloxacin; LVX, levofloxacin; KAN, kanamycin; ETH, ethionamide.

### MIRU-VNTR genotype profiles of the Mtb isolates

**Figure 1:**
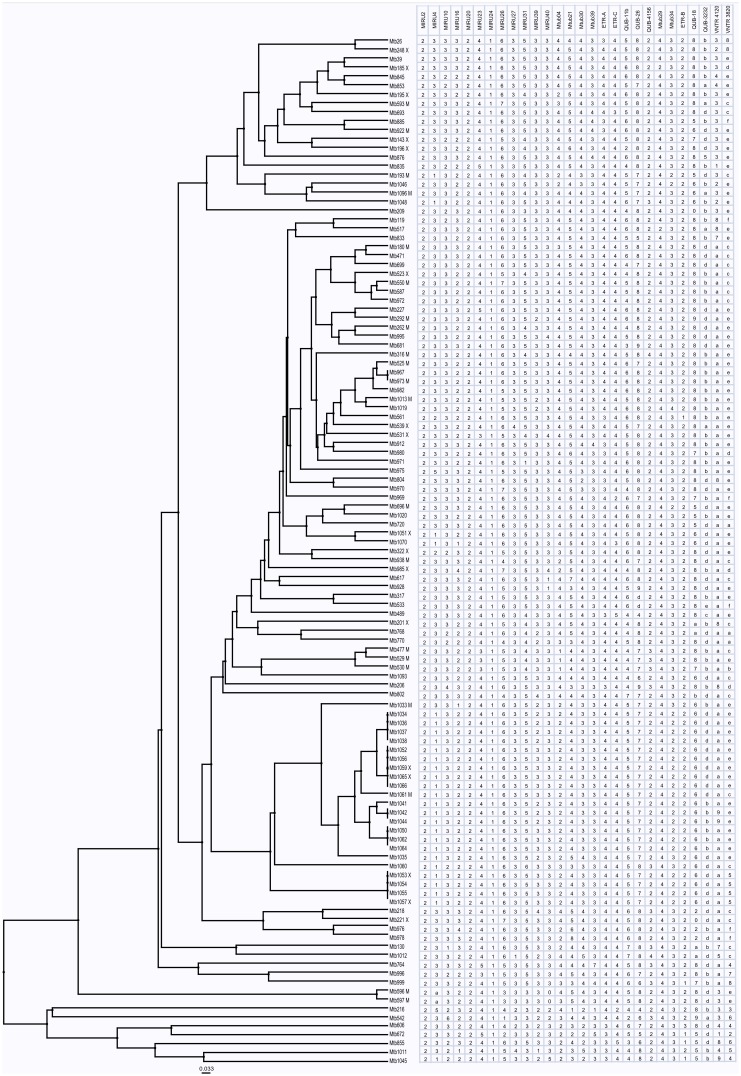
UPGMA tree based on the 28 loci MIRU-VNTR data of 115 *M. tuberculosis* isolates. Note: M = MDR; X = XDR.

Initially, we applied the DTM PCR method to identify the Beijing genotype strains, and the majority of the studied isolates belonged to Beijing genotype (96.5%, 111/115), which limits the usefulness of spoligotyping. We thus further performed 28 loci MIRU-VNTR analyses to genotype all the isolates. The detailed information on the PCR primers used as well as the 28 loci MIRU-VNTR genotype profiles for each isolates are listed in Table S2 and Table S5, respectively. A total of 101 MIRU-VNTR genotypes were detected. Twenty isolates (17.4%) were clustered and 95 isolates (82.6%) had unique genotypes (Fig. 1 and Table S6).

### Association between clinical characteristics of the patients and MIRU-VNTR genotype clustering

Comparison of demographic and clinical characteristics of the patients with clustered vs. unique genotype patterns are shown in Table 4. We observed higher proportion of re-treatment cases among patients with clustered isolates than those with unique MIRU-VNTR genotypes (75.0% vs. 41.1%). However, there was no obvious association between the drug resistance as well as several other characteristics of patients (such as gender, age, underlying diseases, and geographic location, etc.) with the clustered MIRU-VNTR genotypes of the corresponding Mtb isolates. By further examining the clinical data of the clustered isolates, we noticed that two groups of clustered isolates had clinical epidemiological links. Specifically, Mtb 1056 (susceptible) and 1059 (XDR) were isolated from patients having overlapping hospitalization dates in Tuberculosis ward 2, while Mtb 1053 (XDR) and 1057 (XDR) were obtained from patients having overlapping hospitalization dates in Tuberculosis ward 3. The more detailed epidemiological and clinical information of the patients from whom those clustered isolates were isolated were shown in Supplementary Table S7.

**Table 4:** Demographic and clinical characteristics of the patients for clustered vs. unique patterns.

Characteristics	Clusteredn = 20 (%)	Unique patternsn = 95 (%)	*P* value
**Gender**			0.132
Male	9 (45.0)	60 (63.2)	
Female	11 (55.0)	35 (36.8)	
**Age group, years**			0.722
<14	0	1 (1.1)	
15–29	6 (30.0)	38 (40.0)	
30–44	5 (25.0)	20 (21.1)	
45–59	4 (20.0)	15 (15.8)	
60–74	3 (15.0)	18 (18.9)	
>75	2 (10.0)	3 (3.2)	
**Marital status**			0.554
Married	16 (80.0)	70 (73.7)	
Single	4 (20.0)	25 (26.3)	
**Residence situation**			0.623
Beijing resident	5 (25.0)	29 (30.5)	
Migrant	15 (75.0)	66 (69.5)	
**Ethnicity**			0.210
The largest group (Han)	20 (100.0)	88 (92.6)	
Ethnic groups	0	7 (7.4)	
**Geographic location**			0.295
East China	2 (10.0)	7 (7.4)	
South China	0	0	
North China	11 (55.0)	72 (75.8)	
Central China	2 (10.0)	4 (4.2)	
Northeast China	4 (20.0)	7 (7.4)	
Southwest China	1 (5.0)	2 (2.1)	
Northwest China	0	3 (3.2)	
**TB treatment history**			0.006
New cases	5 (25.0)	56 (58.9)	
Re-treatment cases	15 (75.0)	39 (41.1)	
**Underlying diseases**			
Diabetes mellitus	4 (20.0)	14 (14.7)	0.556
Hypertension	2 (10.0)	9 (9.5)	0.942
Abnormal liver function	5 (25.0)	12 (12.6)	0.157
Chronic obstructive pulmonary disease	1 (5.0)	4 (4.2)	0.875
**Sites of TB**			0.107
Extrapulmonary TB	0	14 (14.7)	
Pulmonary TB	12 (60.0)	58 (61.1)	
Pulmonary andextrapulmonary TB	8 (40.0)	23 (24.2)	
**Radiological findings at onset**			0.066
Non-cavitary	6 (30.0)	50 (52.6)	
Cavitary disease	14 (70.0)	45 (47.4)	
**Drug resistance types**			0.110
Susceptible	13 (65.0)	40 (42.1)	
MDR	1 (5.0)	20 (21.1)	
XDR	4 (20.0)	13 (13.7)	
Other types	2 (10.0)	22 (23.2)	
**Treatment outcome**			0.083
Cure	15 (75.0)	79 (83.2)	
Died	1 (5.0)	0	
No data	4 (20.0)	16 (16.8)	

### Phylogenetic analysis of Mtb isolates

A total of 38,355 bp covering drug resistance-associated loci in Mtb were used for analysis as described in a previous study [3]. From the UPGMA tree based on the 28 loci MIRU-VNTR data of Mtb isolates, we observed that clustered isolates contained both susceptible and drug-resistant isolates (Fig. 1).

## Discussion

Early detection of drug resistance in Mtb is important for the successful treatment of TB. New tools for rapid and cost-effective diagnosis of drug resistance in Mtb isolates are urgently needed to control drug-resistant TB, especially the more dangerous MDR- and XDR- TB epidemics. Molecular diagnostics could potentially fill this need but require comprehensive information on the type and frequency of specific drug resistance-associated mutations. A recent study by Zhang et al. reported that through sequencing 161 Mtb isolates with a range of drug resistance profiles, they discovered 72 new genes, 28 intergenic regions, 11 non-synonymous SNPs and 10 intergenic region SNPs with strong and consistent associations with drug resistance in Mtb [11]. In this study, we further identified 36 unreported mutations in drug resistance-associated genes in clinical Mtb isolates. Our study adds to the growing body of knowledge on potential drug resistance-associated mutations in Mtb, which could lead to the development of rapid and more comprehensive molecular diagnostic methods for drug-resistant TB.

Some mutations we identified were seen in both susceptible and resistant isolates. For example, Rv1592c T70 (del) (INH), *gidB* S100F (SM), *embC* R927 (EMB), *ethA* Q360 H (ETH), *gidB* E92D (SM), gyrA (S95T) and *gyrA* E21Q (OFX, LVX) etc. were seen in identical or similar prevalence between susceptible and resistant isolates, thus those mutations should not be associated with drug resistance. Instead, they could be considered as reference specific mutations or phylogenetic informative mutations. The relatively low correlation between the phenotypic and genotypic drug resistance profiles for some drugs were consistent with our previous study and also some other studies [3], [12]. Consistent with several previous studies, our observations showed that certain mutations were more frequently identified in resistant isolates vs. susceptible isolates and thus could serve as useful marker for rapid detection of resistance in the clinical Mtb isolates. For example, S315T and R463L in *katG* (for isoniazid resistance); S531L in *rpoB*; K43R and K88R in *rpsL* (for streptomycin resistance); M306V in *embB* (for ethambutol resistance); D94G and A90V in *gyrA* (for fluoroquinolone resistance); and G1332A and A1401G in *rrs* (for kanamycin resistance), etc. [13]–[15]. Except for those known drug resistance mutations, our studies also revealed a few more newly identified potential drug resistance mutations. For example, A1081 (del) in *rpoB*, A908C in *rrs*, A119D, A193 and A205 in *gidB,* A28 (del) in *pncA*, and A1789 (del) in *rpoB*. We then performed genetic studies by creating point mutation in the susceptible reference strain H37Rv using the pJV53K system [16] for those newly identified potential drug resistance-associated mutations, but failed to confirm their roles in causing drug resistance (data not shown). Our study further confirmed the notion that the genetic basis of drug resistance is more complex than previously anticipated, and a single point mutation could possibly contribute to but is not efficient for drug resistance [17], [18].

Analysis of VNTR profiles specific to endemic strains in certain settings may be useful to identify the sources of outbreaks and transmission pathways. For example, genotyping analysis of MDR-TB isolates revealed that active transmission of MDR- and XDR-TB is taking place in Portugal, and that the high prevalence of observed XDR-TB is due to the continued transmission of particular genetic clusters [19], [20]. But another recent study from Mexico showed that almost all MDR cases studied were epidemiologically unrelated, indicating that the genetic variations observed among those strains are suggestive of emergence of acquired drug-resistance during the course of treatment [21]. A large proportion of the analyzed cases in our study were primary MDR- or XDR-TB. In addition, the clustered MDR-, and XDR-TB cases among the new cases strongly indicated that those patients had been primarily infected with those highly drug-resistant Mtb strains. The observation that clustered isolates contained both susceptible and drug-resistant isolates in the UPGMA tree suggested that the drug resistance traits of Mtb strains are not conserved within defined strain clusters but rather that individual isolates within each cluster have evolved unique characteristics during long-term antibiotic treatment and complicated microbe-host interaction process. This observation further confirmed the previous notion that drug-resistant Mtb isolates have evolved and acquired different mutations independently [3]. We also observed higher proportion of re-treatment cases among patients with clustered isolates than those with unique MIRU-VNTR genotypes. This indicates that those re-treatment cases could be linked with reactivation or recent transmission of new strains. We did notice that several groups of isolates among clustered isolates had clinical epidemiological links, but since those epidemiologically linked clustered isolates had similar but not identical phenotypic and genotypic drug resistance profiles, thus those data could not support the incidence of patient-to-patient transmission during their hospitalization. A previous study indicated the limitations of using MIRU-VNTR typing and epidemiological investigations to detect transmission of Mtb in high burden settings and suggested that whole-genome sequencing (WGS) could provide better solution for tracing the transmission of Mtb in such settings [22]. Thus WGS could be used in the future to better discriminate the possibility of direct transmission events defined by the MIRU-VNTR typing in this study. Nevertheless, since the clustered isolates have a potential of transmission among patients as suggested by several previous studies [19], [20], [23], thus early detection of those isolates and strengthening of control measures to prevent transmission of them are warranted.

A limitation of this study is a possible sampling bias, which could be caused by the following factors. First, the sampling of the participants was not completely randomized but was based on voluntary participation of the patients and availability of the pure cultures of the isolates. Second, since the sample size is relatively small and some mutations were detected at a relatively low frequency, thus the statistical analysis used to identify potential drug resistance mutations in this study might not be suitable for some mutations. For example, D516V in *rpoB* was previously considered to be a drug resistance mutation [24], while the *P* value for this mutation was higher than 0.05. By contrast, a couple of mutations that were known not associated with drug resistance, such as *gidB* E92D [25] and *gyrA* S95T [26], were found with a *P* value lower than 0.05. Third, the sampling was not continuously done, which could lead to reduced clustering of cases. Nevertheless, this study provided important baseline data that may be used to understand and monitor the molecular epidemiology of Mtb isolates in China. Further studies are warranted to better understand the detailed molecular mechanisms in MDR- and XDR-Mtb isolates.

## Supporting Information

Table S1 **Demographic and clinical characteristics of the patients.**
(DOC)

Table S2 **Primers used in this study.**
(DOC)

Table S3 **Summarization of mutations identified in drug resistance-associated loci in** ***M. tuberculosis*** **isolates.**
(DOC)

Table S4 **Correlation between genotypic and phenotypic drug susceptibility testing results based on identification of reported drug resistance associated gene mutations.**
(DOC)

Table S5 **Drug resistance profiles and 28 loci MIRU-VNTR results of** ***M. tuberculosis*** **isolates.**
(DOC)

Table S6 **MIRU-VNTR fingerprinting results for 20 clustered** ***Mycobacterium tuberculosis*** **isolates.**
(DOC)

Table S7 **Drug resistance profiles and epidemiological information of the clustered isolates.**
(DOC)
