# Supplementary material for: Molecular Epidemiology and Clinical Characteristics of Drug-Resistant Mycobacterium tuberculosis in a Tuberculosis Referral Hospital in China
Source: PLoS One. 2014 Oct 10;9(10):e110209. doi: 10.1371/journal.pone.0110209 (PMC4193878; doi:10.1371/journal.pone.0110209)
Supplement: Table S1 — Demographic and clinical characteristics of the patients. (DOC) [file pone.0110209.s001.doc]

| **Table S1.** Demographic and clinical characteristics of the patients. | | | | | | |
| --- | --- | --- | --- | --- | --- | --- |
| **Characteristics** | **Total**  **n=115 (%)** | **Patients infected with Susceptible isolates**  **n=53 (%)** | **Patients infected with MDR isolates(excluding XDR)**  **n=21 (%)** | **Patients infected with XDR isolates**  **n=17 (%)** | **Patients infected with other types of isolates**  **n= 24 (%)** | ***P* value** |
| **Gender** |  |  |  |  |  | 0.128 |
| Male | 69 (60.0) | 36 (67.9) | 10 (47.6) | 7 (41.2) | 16 (66.7) |  |
| Female | 46 (40.0) | 17 (32.1) | 11 (52.4) | 10 (58.8) | 8 (33.3) |  |
| **Age group, years** |  |  |  |  |  | 0.430 |
| <14 | 1 (0.9) | 1 (1.9) | 0 | 0 | 0 |  |
| 15-29 | 44 (38.3) | 18 (34.0) | 10 (47.6) | 5 (29.4) | 11 (45.8) |  |
| 30-44 | 25 (21.7) | 9 (17.0) | 8 (38.1) | 5 (29.4) | 3 (12.5) |  |
| 45-59 | 19 (16.5) | 9 (17.0) | 1 (4.8) | 5 (29.4) | 4 (16.7) |  |
| 60-74 | 21 (18.3) | 13 (24.5) | 1 (4.8) | 2 (11.8) | 5 (20.8) |  |
| >75 | 5 (4.3) | 3 (5.7) | 1 (4.8) | 0 | 1 (4.2) |  |
| **Marital status** |  |  |  |  |  | 0.978 |
| Married | 86 (74.8) | 40 (75.5) | 16 (76.2) | 12 (70.6) | 18 (75.0) |  |
| Single | 29 (25.2) | 13 (24.5) | 5 (23.8) | 5 (29.4) | 6 (25.0) |  |
| **Residence situation** |  |  |  |  |  | 0.703 |
| Beijing Resident | 58 (50.4) | 25 (47.2) | 13 (61.9) | 8 (47.1) | 12 (50.0) |  |
| Migrant | 57 (49.6) | 28 (52.8) | 8 (38.1) | 9 (52.9) | 12 (50.0) |  |
| **Ethnicity** |  |  |  |  |  | 0.596 |
| The largest group (Han) | 108 (93.9) | 49 (92.5) | 19 (90.5) | 17 (100.0) | 23 (95.8) |  |
| Ethnic groups | 7 (6.1) | 4 (7.5) | 2 (9.5) | 0 | 1 (4.2) |  |
| **Geographic location** |  |  |  |  |  | 0.120 |
| East China | 9 (7.8) | 4 (7.5) | 2 (9.5) | 2 (11.8) | 1 (4.2) |  |
| South China | 0 | 0 | 0 | 0 | 0 |  |
| North China | 83 (72.2) | 39 (73.6) | 17 (81.0) | 8 (47.1) | 19 (79.2) |  |
| Central China | 6 (5.2) | 1 (1.9) | 1 (4.8) | 2 (11.8) | 2 (8.3) |  |
| Northeast China | 11 (9.6) | 7 (6.1) | 0 | 4 (23.5) | 0 |  |
| Southwest China | 3 (2.6) | 0 | 0 | 1 (5.9) | 2 (8.3) |  |
| Northwest China | 3 (2.6) | 2 (3.8) | 1 (4.8) | 0 | 0 |  |
| **TB treatment history** |  |  |  |  |  | 0.318 |
| New cases | 61 (53.0) | 33 (62.3) | 10 (47.6) | 7 (41.2) | 11 (45.8) |  |
| Re-treatment cases | 54 (47.0) | 20 (37.7) | 11 (52.4) | 10 (58.8) | 13 (54.2) |  |
| **Underlying diseases** |  |  |  |  |  |  |
| Diabetes mellitus | 18 (15.4) | 9 (17.0) | 4 (19.0) | 1 (5.9) | 4 (16.7) | 0.682 |
| Hypertension | 11 (9.6) | 6 (5.2) | 1 (4.2) | 1 (5.9) | 3 (2.5) | 0.740 |
| Abnormal liver function | 5 (4.3) | 3 (2.6) | 1 (4.2) | 0 | 1 (4.2) | 0.801 |
| Chronic obstructive pulmonary disease | 17 (14.8) | 8 (7.0) | 0 | 2 (11.8) | 7 (29.2) | 0.052 |
| **Sites of TB** |  |  |  |  |  | 0.516 |
| Extrapulmonary TB | 14 (12.2) | 6 (11.3) | 3 (14.3) | 1 (5.9) | 4 (16.7) |  |
| Pulmonary TB | 70 (60.9) | 33 (62.3) | 11 (52.4) | 14 (82.4) | 12 (50.0) |  |
| Pulmonary and extrapulmonary TB | 31 (27.0) | 14 (26.4) | 7 (33.3) | 2 (11.8) | 8 (33.3) |  |
| **Radiological findings at onset** |  |  |  |  |  | 0.322 |
| Non-cavitary | 56 (48.7) | 29 (54.7) | 11 (52.4) | 5 (29.4) | 11 (45.8) |  |
| Cavitary disease | 59 (51.3) | 24 (45.3) | 10 (47.6) | 12 (70.6) | 13 (54.2) |  |
| **Treatment outcome** |  |  |  |  |  | 0.939 |
| Cure | 94 (81.7) | 43 (81.1) | 18 (85.7) | 13 (76.5) | 20 (83.3) |  |
| Died | 1 (0.9) | 1 (1.9) | 0 | 0 | 0 |  |
| No data | 20 (17.4) | 9 (17.0) | 3 (14.3) | 4 (23.5) | 4 (16.7) |  |
|  | | | | | | |
