# Supplementary material for: Molecular Epidemiology and Clinical Characteristics of Drug-Resistant Mycobacterium tuberculosis in a Tuberculosis Referral Hospital in China
Source: PLoS One. 2014 Oct 10;9(10):e110209. doi: 10.1371/journal.pone.0110209 (PMC4193878; doi:10.1371/journal.pone.0110209)
Supplement: Table S2 — Primers used in this study. (DOC) [file pone.0110209.s002.doc]

| **Table S2.** Primers used in this study. | | | | |
| --- | --- | --- | --- | --- |
| **No./Alias** | Primer pairs (5’ to 3’) | | **Reference^a^** | |
| **Primers used for amplifying and sequencing for 45 drug resistance-associated loci** | | | | |
| 1 | | TGTGAACAAGGCTGTGTCCT | |  |
|  | | TTTCCCTCAGCATCTCCATC | |  |
| 2 | | CGAGTTAGCTTGGCTTCCAG | |  |
|  | | TATCCAGCATGTGCTCAACG | |  |
| 3 | | CGATGCGTCGTATGCTTG | |  |
|  | | ATCACGGCAGTAAAACCCAG | |  |
| 4 | | CTGGGTTTTACTGCCGTGAT | |  |
|  | | CGACAGATGAGGCATAGCAG | |  |
| 5 | | GTACGGCCTTTTCGGACTAC | |  |
|  | | CTCCAAATCCGAAGCAAGTC | |  |
| 6 | | CCGGGGCCATAA AATGATTA | |  |
|  | | GCAGGTCGAAACTCAGCC | |  |
| 7 | | CATCGACGCGATGCTCTC | |  |
|  | | GCAATGACGTACGGGGTC | |  |
| 8 | | GGTGTTCATGGGTGACTTCC | |  |
|  | | AGCCCG AGCTTCTTGTTGAC | |  |
| 9 | | CATCGAATATCTGGTCCGCT | |  |
|  | | GTCGTCAGTACAGGGACCGT | |  |
| 10 | | CATGGCCGACAAACAGAAC | |  |
|  | | GTGGCATCAGCCCTTCTCT | |  |
| 11 | | GTAGGGCCCAAACATCGAG | |  |
|  | | GACACCCTATTGGCACCTTG | |  |
| 12 | | GGTGAGTAACACGTGGGTG | |  |
|  | | AGTGTGTTGGTGGCCAACTT | |  |
| 13 | | GACATACCTGCTGCGCAAT | |  |
|  | | GTCACA TTCGACGCCAAAC | |  |
| 14 | | GCCACTGACACAACACAAGG | |  |
|  | | CGCATCCTTCATGTTCCAG | |  |
| 15 | | GGTGTCAGCGCTTGGATT | |  |
|  | | GAACATGTGTCCCTGCACC | |  |
| 16 | | CAGGTGGGAAGTAGAACGGA | |  |
|  | | GGCCATGAAGGGAGCCTA | |  |
| 17 | | CTCTGGCCGAACTCGAAG | |  |
|  | | CAGCAGAACACTGCGATGAG | |  |
| 18 | | AGATCATGGACGACTACGCC | |  |
|  | | TATTTCGAAGACCGGCAATC | |  |
| 19 | | CATCGATCCCTTGTCGAAGT | |  |
|  | | ACACATGTCCTCAACTGGGC | |  |
| 20 | | CTTTCTCCTCATCCCCGTC | |  |
|  | | CGAGATCCTGTACGGCTACG | |  |
| 21 | | CTCATAGATCGGATCCACCC | |  |
|  | | ATCGGAACATACGAAGGCTG | |  |
| 22 | | GTGAACAACCCGACCCAG | |  |
|  | | GGCGTCATGGACCCTATATC | |  |
| 23 | | GTTCGGCTTTTCCGACACT | |  |
|  | | GCACTGTGTTTGGATGAACG | |  |
| 24 | | GTCTCCCAGCTCACCCAAC | |  |
|  | | AGTCCGCGGTTATAGGTTTG | |  |
| 25 | | CACGTGGAAGGGTCTGTTG | |  |
|  | | CTTCCATATCGGTCCGACTC | |  |
| 26 | | ACGACGAACTCGATGCCTAC | |  |
|  | | AACTCGTCGAACGGTGTGTC | |  |
| 27 | | CTGGCCAGGTAAGACGACC | |  |
|  | | TTCTCGATATGAGCCAGTCG | |  |
| 28 | | TGAACCACTGCTTTGCCG | |  |
|  | | TGATGTCTTTGGCGTACTCG | |  |
| 29 | | ACACGCGTCACTCCTTGATT | |  |
|  | | TCTTCCAGTTCTACGTCGCC | |  |
| 30 | | CCACACCCTGGGACAACC | |  |
|  | | TATGACGGCTCTCAACGACA | |  |
| 31 | | TTTCGATGCTGTTTGTCAGC | |  |
|  | | TGCTGTCGATCTCCAATGAG | |  |
| 32 | | TATCAGATGCCTGGCGTGTA | |  |
|  | | AGGTGGTGTTGGTCATACGG | |  |
| 33 | | CAACGCACCGGGAGATAC | |  |
|  | | ATGGTGTTTAACGGCGATGT | |  |
| 34 | | AGTAGTGCATCGATACTGCCC | |  |
|  | | TACGAACCGTACGAACCACA | |  |
| 35 | | GGCAAGGCGGATCTTCTC | |  |
|  | | CGACGACCTCAGTCCACAG | |  |
| 36 | | GTTTCGTCGTCGAGGACATT | |  |
|  | | AAGGCACCGATGATGCAG | |  |
| 37 | | CTAGCCGGAATTGTCCAGTC | |  |
|  | | GTCTCGTCGACGCCGTAT | |  |
| 38 | | AACCTAGGAACGGTGACTCG | |  |
|  | | GTTGTTGAACGGCAGCCAG | |  |
| 39 | | GGTCACGTGGGTGTTGGT | |  |
|  | | ATTGCTCAGGGCTCAGGTT | |  |
| 40 | | AGGCAAGGACGCCAATTT | |  |
|  | | GTTCTCGGTATACCACGCCT | |  |
| 41 | | GTCATCCTGACCGTGGTGTT | |  |
|  | | GGCTGAGGAGACTTTTGTGG | |  |
| 42 | | GCTAAAGCTAAACCCCCACC | |  |
|  | | CGACTATCGACGGACAACAA | |  |
| 43 | | TCGTGGTGATGTCCACTTGT | |  |
|  | | CGCTAAGAACATCGTCGTGA | |  |
| 44 | | TCACGACGATGTTCTTAGCG | |  |
|  | | GGCCTCGACATTACGTTGAT | |  |
| 45 | | GATAGTTGAAGCCTGGCCC | |  |
|  | | GGA GTG CGT AAT GTC TCC GA | |  |
| | **Primers used for 28 loci MIRU-VNTR analysis** | | --- | | | | | |
| MIRU2 | | TGGACTTGCAGCAATGGACCAACT | |  |
|  | | TACTCGGACGCCGGCTCAAAAT | |  |
| MIRU4 | | GCGCGAGAGCCCGAACTGC | |  |
|  | | GCGCAGCAGAAACGTCAGC | |  |
| MIRU10 | | GTTCTTGACCAACTGCAGTCGTCC | |  |
|  | | GCCACCTTGGTGATCAGCTACCT | |  |
| MIRU16 | | TCGGTGATCGGGTCCAGTCCAAGTA | |  |
|  | | CCCGTCGTGCAGCCCTGGTAC | |  |
| MIRU20 | | TCGGAGAGATGCCCTTCGAGTTAG | |  |
|  | | GGAGACCGCGACCAGGTACTTGTA | |  |
| MIRU23 | | CAGCGAAACGAACTGTGCTATCAC | |  |
|  | | CGTGTCCGAGCAGAAAAGGGTAT | |  |
| MIRU24 | | CGACCAAGATGTGCAGGAATACAT | |  |
|  | | GGGCGAGTTGAGCTCACAGAA | |  |
| MIRU26 | | CCCGCCTTCGAAACGTCGCT | |  |
|  | | TGGACATAGGCGACCAGGCGAATA | |  |
| MIRU27 | | TCGAAAGCCTCTGCGTGCCAGTAA | |  |
|  | | GCGATGTGAGCGTGCCACTCAA | |  |
| MIRU31 | | ACTGATTGGCTTCATACGGCTTTA | |  |
|  | | GTGCCGACGTGGTCTTGAT | |  |
| MIRU39 | | CGCATCGACAAACTGGAGCCAAAC | |  |
|  | | CGGAAACGTCTACGCCCCACACAT | |  |
| MIRU40 | | GGGTTGCTGGATGACAACGTGT | |  |
|  | | GGGTGATCTCGGCGAAATCAGATA | |  |
| ETR-A | | AAATCGGTCCCATCACCTTCTTAT | |  |
|  | | CGAAGCCTGGGGTGCCCGCGATTT | |  |
| ETR-B | | ATGGCCACCCGATACCGCTTCAGT | |  |
|  | | CGACGGGCCATCTTGGATCAGCTAC | |  |
| ETR-C | | CGAGAGTGGCAGTGGCGGTTATCT | |  |
|  | | AATGACTTGAACGCGCAAATTGTGA | |  |
| Mtub04 | | CTTGGCCGGCATCAAGCGCATTATT | |  |
|  | | GGCAGCAGAGCCCGGGATTCTTC | |  |
| Mtub21 | | AGATCCCAGTTGTCGTCGTC | |  |
|  | | CAACATCGCCTGGTTCTGT A | |  |
| Mtub29 | | GCCAGCCGCCGTGCATAAACCT | |  |
|  | | AGCCACCCGGTGTGCCTTGTATGAC | |  |
| Mtub30 | | CTTGAAGCCCCGGTCTCATCTGT | |  |
|  | | ACTTGAACCCCCACGCCCATTAGTA | |  |
| Mtub34 | | GGTGCGCACCTGCTCCAGATAA | |  |
|  | | GGCTCTCATTGCTGGAGGGTTGTAC | |  |
| Mtub39 | | CGGTGGAGGCGATGAACGTCTTC | |  |
|  | | AGAGCGGCACGGGGGAAAGCTTAG | |  |
| QUB-11b | | CGTAAGGGGGATGCGGGAAATAGG | |  |
|  | | CGAAGTGAATGGTGGCAT | |  |
| QUB-26 | | AACGCTCAGCTGTCGGAT | |  |
|  | | CCGTGCCGGCCAGGTCCTTCCCGAT | |  |
| QUB-4156 | | TGACCACGGATTGCTCTAGT | |  |
|  | | GCCGGCGTCCATGTT | |  |
| QUB-18 | | ATCGTCAGCTGCGGAATAGT | |  |
|  | | AATACCGGGGATATCGGTTC | |  |
| QUB-3232 | | CAGACCCGGCGTCATCAAC | |  |
|  | | CCAAGGGCGGCATTGTGTT | |  |
| VNTR 4120 | | GTTCACCGGAGCCAACC | |  |
|  | | GAGGTGGTTTCGTGGTCG | |  |
| VNTR 3820 | | TGCGCGGTGAATGAGACG | |  |
|  | | ACCTTCATCCTTGGCGAC | |  |
| ***^a^*** See the references bellow.  1. Liu CH, Li HM, Lu N, Wang Q, Hu YL, et al. (2012) Genomic sequence based scanning for drug resistance-associated mutations and evolutionary analysis of multidrug-resistant and extensively drug-resistant Mycobacterium tuberculosis. J Infect 65: 412-422.  2. Supply P, Allix C, Lesjean S, Cardoso-Oelemann M, Rusch-Gerdes S, et al. (2006) Proposal for standardization of optimized mycobacterial interspersed repetitive unit-variable-number tandem repeat typing of Mycobacterium tuberculosis. J Clin Microbiol 44: 4498-4510.  3. Skuce RA, McCorry TP, McCarroll JF, Roring SMM, Scott AN, et al. (2002) Discrimination of Mycobacterium tuberculosis complex bacteria using novel VNTR-PCR targets. Microbiology-Sgm 148: 519-528.  4. Roring S, Scott A, Brittain D, Walker I, Hewinson G, et al. (2002) Development of variable-number tandem repeat typing of Mycobacterium bovis: Comparison of results with those obtained by using existing exact tandem repeats and spoligotyping. Journal of Clinical Microbiology 40: 2126-2133.  5. Smittipat N, Billamas P, Palittapongarnpim M, Thong-On A, Temu MM, et al. (2005) Polymorphism of variable-number tandem repeats at multiple loci in Mycobacterium tuberculosis. Journal of Clinical Microbiology 43: 5034-5043. | | | | |
