# Supplementary material for: Molecular Epidemiology and Clinical Characteristics of Drug-Resistant Mycobacterium tuberculosis in a Tuberculosis Referral Hospital in China
Source: PLoS One. 2014 Oct 10;9(10):e110209. doi: 10.1371/journal.pone.0110209 (PMC4193878; doi:10.1371/journal.pone.0110209)
Supplement: Table S3 — Summarization of mutations identified in drug resistance-associated loci in M. tuberculosis isolates. (DOC) [file pone.0110209.s003.doc]

| **Table S3.** Summarization of mutations identified in drug resistance-associated loci in *M. tuberculosis* isolates. | | | | | | | | | |
| --- | --- | --- | --- | --- | --- | --- | --- | --- | --- |
| **Mutations in target gene or intergenic regions (corresponding drugs)^a^** | | | | | | | | | |
| **Isolates** | **Rv0340 (INH)** | **Rv0341 (*iniB*)**  **(INH)** | **Rv1483 (*mabA*)^b^ (INH)** | **Rv1592c (INH)** | **Rv1908c (*katG*) (INH)** | **Rv2247 (*accD6*) (INH)** | **Rv2428 (*ahpC*)^b^ (INH)** | **Rv2846c (*efpA*)**  **(INH)** | **Rv0667 (*rpoB*)^c^ (RMP)** |
| 26 | None (S) | None (S) | None (S) | T70 (del) (S) | None (S) | None (S) | None (S) | None (S) | None (S) |
| 39 | None (S) | None (S) | None (S) | T70 (del) (S) | None (S) | None (S) | None (S) | None (S) | None (S) |
| 143 | None (R) | None (R) | T-8C^f^ (R) | T70 (del) (R) | S315T^f,g^,R463L^g^ (R) | D229G^g^ (R) | None (R) | None (R) | S531L^f,g^ (R) |
| 119 | None (R) | None (R) | None (R) | T70 (del) (R) | R463L^g^ (R) | D229G^g^ (R) | None (R) | None (R) | None (S) |
| 130 | None (S) | None (S) | None (S) | T70 (del) (S) | None (S) | None (S) | None (S) | None (S) | None (S) |
| 180 | None (R) | None (R) | None (R) | T70 (del) (R) | R463L^g^ (R) | D229G^g^ (R) | None (R) | None (R) | None (R) |
| 185 | None (R) | None (R) | None (R) | T70 (del) (R) | R463L^g^ (R) | D229G^g^ (R) | None (R) | None (R) | H526D^f,g^ (R) |
| 193 | None (R) | None (R) | None (R) | T70 (del) (R) | R463L^g^ (R) | D229G^g^ (R) | None (R) | None (R) | None (R) |
| 195 | None (R) | None (R) | None (R) | T70 (del) (R) | R463L^g^ (R) | D229G^g^ (R) | None (R) | None (R) | None (R) |
| 196 | None (R) | None (R) | None (R) | T70 (del) (R) | R463L^g^ (R) | D229G^g^ (R) | None (R) | None (R) | None (R) |
| 201 | G148^d^ (R) | None (R) | None (R) | T70 (del) (R) | R463L^g^ (R) | D229G^g^ (R) | G-48A (R) | None (R) | H526D^f,g^ (R) |
| 206 | None (R) | None (R) | None (R) | T70 (del) (R) | S315T^f,g^,R463L^g^ (R) | D229G^g^ (R) | None (R) | None (R) | None (S) |
| 209 | None (R) | None (R) | None (R) | T70 (del) (R) | R463L^g^ (R) | D229G^g^ (R) | None (R) | None (R) | None (S) |
| 216 | None (R) | None (R) | None (R) | T70 (del) (R) | None (R) | None (R) | None (R) | None (R) | None (S) |
| 218 | None (R) | None (R) | None (R) | T70 (del) (R) | R463L^g^ (R) | D229G^g^ (R) | None (R) | None (R) | None (S) |
| 221 | None (R) | None (R) | None (R) | T70 (del) (R) | C189G^e,g^,R463L^g^ (R) | D229G^g^ (R) | None (R) | None (R) | S531L^f,g^ (R) |
| 227 | None (S) | None (S) | None (S) | T70 (del) (S) | None (S) | None (S) | None (S) | None (S) | None (S) |
| 248 | None (R) | None (R) | None (R) | T70 (del) (R) | S315T^f,g^ (R) | None (R) | T249^d,e^,D253^d,e^,S221T^e,g^,A254D^e,g^(R) | None (R) | D516V ^f,g^ (R) |
| 262 | None (R) | None (R) | None (R) | T70 (del) (R) | S315T^f,g^,R463L^g^ (R) | D229G^g^ (R) | None (R) | None (R) | S531L^f,g^ (R) |
| 292 | None (R) | None (R) | None (R) | T70 (del) (R) | D171A^e,g^,R463L^g^ (R) | D229G^g^ (R) | None (R) | None (R) | None (R) |
| 316 | None (R) | None (R) | None (R) | T70 (del) (R) | R463L^g^ (R) | D229G^g^ (R) | None (R) | T368(del)(R) | S531L^f,g^ (R) |
| 317 | None (S) | None (S) | None (S) | T70 (del) (S) | None (S) | None (S) | None (S) | None (S) | None (S) |
| 322 | None (R) | None (R) | None (R) | T70 (del) (R) | R463L^g^ (R) | D229G^g^ (R) | None (R) | None (R) | None (R) |
| 471 | None (S) | None (S) | None (S) | T70 (del) (S) | None (S) | None (S) | None (S) | None (S) | None (S) |
| 477 | None (R) | CTGGTGTCGGCG665 (del) (R) | None (R) | T70 (del) (R) | S315T^f,g^,R463L^g^ (R) | D229G^g^ (R) | None (R) | None (R) | S531L^f,g^ (R) |
| 489 | None (S) | None (S) | None (S) | T70 (del) (S) | None (S) | None (S) | None (S) | None (S) | None (S) |
| 517 | None (R) | None (R) | None (R) | T70 (del) (R) | R463L^g^ (R) | D229G^g^ (R) | None (R) | None (R) | None (S) |
| 523 | None (R) | None (R) | None (R) | T70 (del) (R) | S315T^f,g^,R463L^g^ (R) | D229G^g^ (R) | None (R) | None (R) | S531L^f,g^ (R) |
| 525 | None (R) | None (R) | None (R) | T70 (del) (R) | S315T^f,g^,R463L^g^ (R) | D229G^g^ (R) | S40N^e,g^ (R) | None (R) | L511P^f,g^,A1789(del)^e^ (R) |
| 529 | None (R) | CTGGTGTCGGCG665 (del) (R) | None (R) | T70 (del) (R) | S315T^f,g^,R463L^g^ (R) | D229G^g^ (R) | None (R) | None (R) | S531L^f,g^ (R) |
| 530 | None (R) | CTGGTGTCGGCG665 (del) (R) | None (R) | T70 (del) (R) | S315T^f,g^,R463L^g^ (R) | D229G^g^ (R) | None (R) | None (R) | S531L^f,g^ (R) |
| 531 | None (R) | None (R) | None (R) | T70 (del) (R) | R463L^g^ (R) | D229G^g^ (R) | C-81T (R) | None (R) | H526D^f,g^ (R) |
| 533 | None (S) | None (S) | None (S) | T70 (del) (S) | None (S) | None (S) | None (S) | None (S) | None (S) |
| 539 | None (R) | None (R) | None (R) | T70 (del) (R) | R463L^g^ (R) | D229G^g^ (R) | None (R) | None (R) | A1081(del)^e^ (R) |
| 542 | None (R) | None (R) | C-15T (R) | T70 (del) (R) | None (R) | None (R) | None (R) | None (R) | None (S) |
| 550 | None (R) | None (R) | None (R) | T70 (del) (R) | R463L^g^ (R) | D229G^g^ (R) | None (R) | None (R) | A1081(del)^e^ (R) |
| 561 | None (S) | None (S) | None (S) | T70 (del) (S) | None (S) | None (S) | None (S) | None (S) | None (S) |
| 587 | None (S) | None (S) | None (S) | T70 (del) (S) | None (S) | None (S) | None (S) | None (S) | None (S) |
| 593 | None (R) | None (R) | None (R) | T70 (del) (R) | R463L^g^ (R) | D229G^g^ (R) | None (R) | None (R) | None (R) |
| 596 | None (R) | None (R) | None (R) | T70 (del) (R) | None (R) | None (R) | None (R) | None (R) | None (R) |
| 597 | None (R) | None (R) | None (R) | T70 (del) (R) | R463L^g^ (R) | D229G^g^ (R) | None (R) | None (R) | None (R) |
| 606 | None (S) | None (S) | None (S) | T70 (del) (S) | None (S) | None (S) | None (S) | None (S) | None (S) |
| 617 | None (S) | None (S) | None (S) | T70 (del) (S) | None (S) | None (S) | None (S) | None (S) | None (S) |
| 672 | None (S) | None (S) | None (S) | T70 (del) (S) | None (S) | None (S) | None (S) | None (S) | None (S) |
| 681 | None (S) | None (S) | None (S) | T70 (del) (S) | None (S) | None (S) | None (S) | None (S) | None (S) |
| 693 | None (S) | None (S) | None (S) | T70 (del) (S) | None (S) | None (S) | None (S) | None (S) | None (S) |
| 696 | None (R) | None (R) | None (R) | T70 (del) (R) | R463L^g^ (R) | D229G^g^ (R) | None (R) | None (R) | None (R) |
| 699 | None (S) | None (S) | None (S) | T70 (del) (S) | None (S) | None (S) | None (S) | None (S) | None (S) |
| 720 | None (S) | None (S) | None (S) | T70 (del) (S) | None (S) | None (S) | None (S) | None (S) | T481A^e,g^ (R) |
| 764 | None (S) | None (S) | None (S) | T70 (del) (S) | None (S) | None (S) | None (S) | None (S) | None (S) |
| 768 | None (S) | None (S) | None (S) | T70 (del) (S) | None (S) | None (S) | None (S) | None (S) | None (S) |
| 770 | None (S) | None (S) | None (S) | T70 (del) (S) | None (S) | None (S) | None (S) | None (S) | None (S) |
| 802 | None (S) | None (S) | None (S) | T70 (del) (S) | None (S) | None (S) | None (S) | None (S) | None (S) |
| 804 | None (S) | None (S) | None (S) | T70 (del) (S) | None (S) | None (S) | None (S) | None (S) | None (S) |
| 833 | None (S) | None (S) | None (S) | T70 (del) (S) | None (S) | None (S) | None (S) | None (S) | None (S) |
| 835 | None (S) | None (S) | None (S) | T70 (del) (S) | None (S) | None (S) | None (S) | None (S) | None (S) |
| 845 | None (S) | None (S) | None (S) | T70 (del) (S) | None (S) | None (S) | None (S) | None (S) | None (S) |
| 853 | None (S) | None (S) | None (S) | T70 (del) (S) | None (S) | None (S) | None (S) | None (S) | None (S) |
| 855 | None (S) | None (S) | None (S) | T70 (del) (S) | None (S) | None (S) | None (S) | None (S) | None (S) |
| 876 | None (S) | None (S) | None (S) | T70 (del) (S) | None (S) | None (S) | None (S) | None (S) | None (S) |
| 885 | None (S) | None (S) | None (S) | T70 (del) (S) | None (S) | None (S) | None (S) | None (S) | None (S) |
| 912 | None (S) | None (S) | None (S) | T70 (del) (S) | None (S) | None (S) | None (S) | None (S) | None (S) |
| 922 | None (R) | None (R) | None (R) | T70 (del) (R) | S315T^f,g^,R463L^g^ (R) | D229G^g^ (R) | None (R) | None (R) | S531L^f,g^ (R) |
| 928 | None (S) | None (S) | None (S) | T70 (del) (S) | None (S) | None (S) | None (S) | None (S) | None (S) |
| 938 | None (R) | None (R) | None (R) | T70 (del) (R) | R463L^g^ (R) | D229G^g^ (R) | None (R) | None (R) | S531L^f,g^ (R) |
| 967 | None (S) | None (S) | None (S) | T70 (del) (S) | None (S) | None (S) | None (S) | None (S) | None (S) |
| 969 | None (S) | None (S) | None (S) | T70 (del) (S) | None (S) | None (S) | None (S) | None (S) | None (S) |
| 970 | None (R) | None (R) | None (R) | T70 (del) (R) | S315T^f,g^,R463L^g^ (R) | D229G^g^ (R) | None (R) | None (R) | None (S) |
| 971 | None (S) | None (S) | None (S) | T70 (del) (S) | None (S) | None (S) | None (S) | None (S) | None (S) |
| 972 | None (S) | None (S) | None (S) | T70 (del) (S) | None (S) | None (S) | None (S) | None (S) | None (S) |
| 973 | None (R) | None (R) | None (R) | T70 (del) (R) | R463L^g^ (R) | D229G^g^ (R) | C-52T (R) | None (R) | D516A^f,g^,H526R^f,g^ **R688S**^e,g^,A1789(del)^e^ (R) |
| 975 | None (S) | None (S) | None (S) | T70 (del) (S) | None (S) | None (S) | None (S) | None (S) | None (S) |
| 976 | None (S) | None (S) | None (S) | T70 (del) (S) | None (S) | None (S) | None (S) | None (S) | None (S) |
| 978 | None (S) | None (S) | None (S) | T70 (del) (S) | None (S) | None (S) | None (S) | None (S) | None (S) |
| 980 | None (S) | None (S) | None (S) | T70 (del) (S) | None (S) | None (S) | None (S) | None (S) | None (S) |
| 982 | None (S) | None (S) | None (S) | T70 (del) (S) | None (S) | None (S) | None (S) | None (S) | None (S) |
| 985 | None (R) | None (R) | T-8C^f^ (R) | T70 (del) (R) | S315T^f,g^,R463L^g^ (R) | D229G^g^ (R) | None (R) | None (R) | L511P^f,g^,S512G^f,g^,D516G^f,g^,A1789(del)^e^ (R) |
| 995 | None (S) | None (S) | None (S) | T70 (del) (S) | None (S) | None (S) | None (S) | None (S) | None (S) |
| 996 | None (S) | None (S) | None (S) | T70 (del) (S) | None (S) | None (S) | None (S) | None (S) | None (S) |
| 999 | None (S) | None (S) | None (S) | T70 (del) (S) | None (S) | None (S) | None (S) | None (S) | None (S) |
| 1011 | None (S) | None (S) | None (S) | T70 (del) (S) | None (S) | None (S) | None (S) | None (S) | None (S) |
| 1012 | None (S) | None (S) | None (S) | T70 (del) (S) | None (S) | None (S) | None (S) | None (S) | None (S) |
| 1013 | None (R) | None (R) | None (R) | T70 (del) (R) | S315T^f,g^,R463L^g^ (R) | D229G^g^ (R) | None (R) | None (R) | A1789(del)^e^ (R) |
| 1019 | None (S) | None (S) | None (S) | T70 (del) (S) | None (S) | None (S) | None (S) | None (S) | None (S) |
| 1020 | None (S) | None (S) | None (S) | T70 (del) (S) | None (S) | None (S) | None (S) | None (S) | None (S) |
| 1033 | None (R) | None (R) | None (R) | T70 (del) (R) | R463L^g^ (R) | D229G^g^ (R) | None (R) | None (R) | S531L^f,g^ (R) |
| 1034 | None (S) | None (S) | None (S) | T70 (del) (S) | None (S) | None (S) | None (S) | None (S) | None (S) |
| 1035 | None (R) | None (R) | None (R) | T70 (del) (R) | R463L^g^ (R) | D229G^g^ (R) | G-51A (R) | None (R) | None (S) |
| 1036 | None (S) | None (S) | None (S) | T70 (del) (S) | None (S) | None (S) | None (S) | None (S) | None (S) |
| 1037 | None (S) | None (S) | None (S) | T70 (del) (S) | None (S) | None (S) | None (S) | None (S) | None (S) |
| 1038 | None (S) | None (S) | None (S) | T70 (del) (S) | None (S) | None (S) | None (S) | None (S) | None (S) |
| 1041 | None (S) | None (S) | None (S) | T70 (del) (S) | None (S) | None (S) | None (S) | None (S) | None (S) |
| 1042 | None (S) | None (S) | None (S) | T70 (del) (S) | None (S) | None (S) | None (S) | None (S) | None (S) |
| 1044 | None (S) | None (S) | None (S) | T70 (del) (S) | None (S) | None (S) | None (S) | None (S) | None (S) |
| 1045 | None (S) | None (S) | None (S) | T70 (del) (S) | None (S) | None (S) | None (S) | None (S) | None (S) |
| 1046 | None (S) | None (S) | None (S) | T70 (del) (S) | None (S) | None (S) | None (S) | None (S) | None (S) |
| 1048 | None (S) | None (S) | None (S) | T70 (del) (S) | None (S) | None (S) | None (S) | None (S) | None (S) |
| 1050 | None (S) | None (S) | None (S) | T70 (del) (S) | None (S) | None (S) | None (S) | None (S) | None (S) |
| 1051 | None (R) | None (R) | T-8C^f^ (R) | T70 (del) (R) | S315T^f,g^,R463L^g^ (R) | D229G^g^ (R) | None (R) | None (R) | S531L^f,g^ (R) |
| 1052 | None (S) | None (S) | None (S) | T70 (del) (S) | None (S) | None (S) | None (S) | None (S) | None (S) |
| 1053 | None (R) | None (R) | None (R) | T70 (del) (R) | R463L^g^ (R) | D229G^g^ (R) | None (R) | None (R) | None (R) |
| 1054 | None (S) | None (S) | None (S) | T70 (del) (S) | None (S) | None (S) | None (S) | None (S) | None (S) |
| 1055 | None (S) | None (S) | None (S) | T70 (del) (S) | None (S) | None (S) | None (S) | None (S) | None (S) |
| 1056 | None (S) | None (S) | None (S) | T70 (del) (S) | None (S) | None (S) | None (S) | None (S) | None (S) |
| 1057 | None (R) | None (R) | None (R) | T70 (del) (R) | R463L^g^ (R) | D229G^g^ (R) | None (R) | None (R) | None (R) |
| 1059 | None (R) | None (R) | None (R) | T70 (del) (R) | R463L^g^ (R) | D229G^g^ (R) | None (R) | None (R) | A1081(del)^e^ (R) |
| 1060 | None (S) | None (S) | None (S) | T70 (del) (S) | None (S) | None (S) | None (S) | None (S) | None (S) |
| 1061 | None (R) | None (R) | None (R) | T70 (del) (R) | R463L^g^ (R) | D229G^g^ (R) | None (R) | None (R) | None (R) |
| 1062 | None (S) | None (S) | None (S) | T70 (del) (S) | None (S) | None (S) | None (S) | None (S) | None (S) |
| 1064 | None (S) | None (S) | None (S) | T70 (del) (S) | None (S) | None (S) | None (S) | None (S) | None (S) |
| 1065 | None (R) | None (R) | None (R) | T70 (del) (R) | R463L^g^ (R) | D229G^g^ (R) | None (R) | None (R) | None (R) |
| 1066 | None (S) | None (S) | None (S) | T70 (del) (S) | None (S) | None (S) | None (S) | None (S) | None (S) |
| 1070 | None (S) | None (S) | None (S) | T70 (del) (S) | None (S) | None (S) | None (S) | None (S) | None (S) |
| 1093 | None (S) | None (S) | None (S) | T70 (del) (S) | None (S) | None (S) | None (S) | None (S) | None (S) |
| 1096 | None (R) | None (R) | None (R) | T70 (del) (R) | R463L^g^ (R) | D229G^g^ (R) | None (R) | None (R) | None (R) |
| ^a^ “R”, resistance of isolates to the corresponding anti-TB drug; “S”, sensitivity of isolates to the corresponding anti-TB drug; “del”, deletion; “ins”, insertion; INH, isoniazid; RMP, rifampicin; STR, streptomycin; EMB, ethambutol; PZA, pyrazinamide; OFX, ofloxacin; LVX, levofloxacin; KAN, kanamycin; ETH, ethionamide; PAS, para-amino salicylic acid.  ^b^ intergenic regions.  ^c^ nucleotide mutational position is relative to *Mycobacterium tuberculosis* H37Rv *rpoB*, and amino acid position is relative to *Escherichia coli* numbering.  ^d^ synonymous.  ^e^ newly identified mutations.  ^f^ drug resistance-asssociated mutations with high confidence.  ^g^ non-synonymous. | | | | | | | | | |

| **Table S2.** Summarization of mutations identified in drug resistance-associated loci in *M. tuberculosis* isolates (Continued). | | | | | | | | | | | | |
| --- | --- | --- | --- | --- | --- | --- | --- | --- | --- | --- | --- | --- |
| **Mutations in target gene or intergenic regions (corresponding drugs)^a^** | | | | | | | | | | | | |
| **Isolates** | **Rv0682 (*rpsL*) (SM)** | **Rvnr01 (*rrs*) (SM)** | **Rv3919c (*gidB*) (SM)** | **Rv3793 (*embC*) (EMB)** | **Rv3794 (*embA*)^b^ (EMB)** | **Rv3794 (*embA*) (EMB)** | **Rv3795 (*embB*) (EMB)** | **Rv0006 (*gyrA*) (OFX, LVX)** | **Rv2043c (*pncA*) (PZA)** | **Rvnr01 (*rrs*) (KAN)** | **Rv1694 (*tlyA*) (KAN)** | **Rv3854c (*ethA*) (ETH)** |
| 26 | None (S) | None (S) | E92D^g^,S100F^g^,A205^d^ (S) | R927^d^ (S) | None (S) | C76^d^ (S) | None (S) | E21Q^g^,S95T^g^ (S,S) | None (S) | None (S) | L11^d^ (S) | Q360H^g^ (S) |
| 39 | None (S) | None (S) | E92D^g^,S100F^g^,A205^d^ (S) | R927^d^ (S) | None (S) | C76^d^ (S) | None (S) | E21Q^g^,S95T^g^ (S,S) | None (S) | None (S) | L11^d^ (S) | Q360H^g^ (S) |
| 143 | K43R^f,g^ (R) | None (R) | E92D^g^,S100F^g^,A205^d^ (R) | None (R) | None (R) | C76^d^ (R) | M306V^f,g^ (R) | E21Q^g^,D94G^f,g^,S95T^g^ (R,R) | T76I^g^ (R) | G1332A,A1401G (R) | L11^d^ (R) | Q360H^g^ (R) |
| 119 | None (S) | None (S) | E92D^g^,S100F^g^ (S) | R927^d^ (S) | None (S) | C76^d^ (S) | None (S) | E21Q^g^,S95T^g^ (S,S) | None (S) | None (S) | L11^d^ (S) | Q360H^g^ (S) |
| 130 | None (R) | None (R) | E92D^g^,S100F^g^,A205^d^ (R) | R927^d^ (S) | None (S) | C76^d^ (S) | None (S) | E21Q^g^,S95T^g^ (S,S) | None (S) | None (S) | L11^d^ (S) | Q360H^g^ (S) |
| 180 | None (S) | None (S) | E92D^g^,S100F^g^,A205^d^ (S) | R927^d^ (R) | None (R) | C76^d^ (R) | None (R) | E21Q^g^,S95T^g^ (S,S) | None (S) | None (S) | L11^d^ (S) | Q360H^g^ (S) |
| 185 | None (R) | None (R) | E92D^g^,S100F^g^,A205^d^ (R) | R927^d^ (R) | None (R) | C76^d^ (R) | M306V^f,g^ (R) | E21Q^g^,A90V^f,g^,S95T^g^ (R,R) | None (R) | None (R) | L11^d^ (R) | Q360H^g^ (S) |
| 193 | None (R) | None (R) | E92D^g^,S100F^g^,A205^d^ (R) | R927^d^ (R) | None (R) | C76^d^ (R) | None (R) | E21Q^g^,S95T^g^ (R,R) | None (S) | None (S) | L11^d^ (S) | Q360H^g^ (S) |
| 195 | None (R) | None (R) | E92D^g^,S100F^g^,A205^d^ (R) | R927^d^ (S) | None (S) | C76^d^ (S) | P195L^g^ (S) | E21Q^g^,S95T^g^ (R,R) | None (S) | G1332A (R) | L11^d^ (R) | Q360H^g^ (S) |
| 196 | K43R^f,g^ (R) | None (R) | E92D^g^,S100F^g^,A205^d^ (R) | R927^d^ (S) | None (S) | C76^d^ (S) | None (S) | E21Q^g^,S95T^g^ (R,R) | None (S) | None (R) | L11^d^ (R) | Q360H^g^ (S) |
| 201 | K43R^f,g^ (R) | None (R) | E92D^g^,S100F^g^,A205^d^ (R) | R927^d^ (R) | None (R) | C76^d^ (R) | M306V^f,g^ (R) | E21Q^g^,S95T^g^ (R,R) | None (S) | G1332A (R) | L11^d^ (R) | Q360H^g^ (S) |
| 206 | K43R^f,g^ (R) | None (R) | E92D^g^,S100F^g^,A205^d^ (R) | R927^d^ (S) | None (S) | C76^d^ (S) | None (S) | E21Q^g^,S95T^g^ (S,S) | None (S) | None (S) | L11^d^ (S) | Q360H^g^ (R) |
| 209 | None (R) | None (R) | E92D^g^,S100F^g^,A205^d^,A138V^g^ (R) | R927^d^ (R) | None (R) | C76^d^ (R) | D534^d,e^(R) | E21Q^g^,S95T^g^ (R,R) | None (S) | None (S) | L11^d^ (S) | Q360H^g^ (S) |
| 216 | None (S) | None (S) | E92D^g^,S100F^g^ (S) | R927^d^ (S) | None (S) | C76^d^ (S) | None (S) | E21Q^g^,S95T^g^ (S,S) | None (S) | None (S) | L11^d^ (S) | Q360H^g^ (S) |
| 218 | None (R) | None (R) | E92D^g^,S100F^g^,A205^d^ (R) | R927^d^ (R) | None (R) | C76^d^ (R) | None (R) | E21Q^g^,S95T^g^ (R,R) | A28(del) ^e^ (R) | None (S) | L11^d^ (S) | Q360H^g^ (S) |
| 221 | None (R) | None (R) | E92D^g^,S100F^g^,A205^d^ (R) | R927^d^ (R) | None (R) | C76^d^ (R) | None (R) | E21Q^g^,D94G^f,g^,S95T^g^ (R,R) | None (S) | None (R) | L11^d^ (R) | Q360H^g^ (R) |
| 227 | None (S) | None (S) | E92D^g^,S100F^g^ (S) | R927^d^ (S) | None (S) | C76^d^ (S) | None (S) | E21Q^g^,S95T^g^ (S,S) | None (S) | None (S) | L11^d^ (S) | Q360H^g^ (S) |
| 248 | None (R) | None (R) | E92D^g^,S100F^g^,A205^d^ (R) | R927^d^ (R) | None (R) | C76^d^ (R) | D328Y^g^ (R) | E21Q^g^,D94N^f,g^,S95T^g^ (R,R) | None (R) | None (R) | L11^d^ (R) | Q360H^g^ (R) |
| 262 | None (R) | None (R) | E92D^g^,S100F^g^,A205^d^ (R) | R927^d^ (R) | C-12T(R) | C76^d^ (R) | I563L^g^ (R) | E21Q^g^,A90V^f,g^,S95T^g^ (R,R) | GG391 (ins)^f^ (R) | None (S) | L11^d^ (S) | Q360H^g^ (S) |
| 292 | None (S) | None (S) | E92D^g^,S100F^g^,A205^d^ (S) | R927^d^ (S) | None (S) | C76^d^ (S) | R471P^g^ (S) | E21Q^g^,S95T^g^ (R,R) | None (S) | None (S) | L11^d^ (S) | Q360H^g^ (R) |
| 316 | None (R) | None (R) | E92D^g^,S100F^g^,A205^d^ (R) | D793^d^,R927^d^ (S) | None (S) | C76^d^ (S) | Q497R^f,g^ (S) | E21Q^g^,S95T^g^ (S,S) | None (S) | None (S) | L11^d^ (S) | Q360H^g^ (S) |
| 317 | K43R^f,g^ (R) | None (R) | E92D^g^,S100F^g^,A205^d^ (R) | R927^d^ (S) | None (S) | C76^d^ (S) | None (S) | E21Q^g^,S95T^g^ (S,S) | None (S) | None (S) | L11^d^ (S) | Q360H^g^ (R) |
| 322 | None (S) | None (S) | E92D^g^,S100F^g^,A205^d^ (S) | R927^d^ (R) | None (R) | C76^d^ (R) | Q497K^g^ (R) | E21Q^g^,S95T^g^ (R,R) | None (R) | None (R) | L11^d^ (R) | Q360H^g^ (S) |
| 471 | None (S) | None (S) | E92D^g^,S100F^g^,A205^d^ (S) | R927^d^ (R) | None (R) | C76^d^ (R) | None (R) | E21Q^g^,S95T^g^ (R,R) | None (R) | None (S) | L11^d^ (S) | Q360H^g^ (S) |
| 477 | None (R) | A908C (R) | E92D^g^,S100F^g^,A119D^g^,A193^d^,A205^d^(R) | R927^d^ (S) | None (S) | C76^d^ (S) | G406A^g^ (S) | E21Q^g^,D94G^f,g^,S95T^g^ (R,R) | V128G^f,g^ (R) | None (S) | L11^d^ (S) | Q360H^g^ (S) |
| 489 | None (S) | None (S) | E92D^g^,S100F^g^ (S) | R927^d^ (S) | None (S) | C76^d^ (S) | None (S) | E21Q^g^,S95T^g^ (S,S) | None (S) | None (S) | L11^d^ (S) | Q360H^g^ (S) |
| 517 | None (S) | None (S) | E92D^g^,S100F^g^,A205^d^ (S) | R927^d^ (S) | None (S) | C76^d^ (S) | None (S) | E21Q^g^,S95T^g^ (R,R) | None (S) | None (S) | L11^d^ (S) | Q360H^g^ (R) |
| 523 | None (R) | None (R) | E92D^g^,S100F^g^,A119D^g^,A193^d^,A205^d^(R) | R927^d^ (R) | None (R) | C76^d^,G350D^g^ (R) | G406A^g^ (R) | E21Q^g^,D94G^f,g^,S95T^g^ (R,R) | GG391(ins)^f^ (R) | None (R) | L11^d^ (R) | Q360H^g^ (S) |
| 525 | K43R^f,g^ (R) | None (R) | E92D^g^,S100F^g^,A205^d^ (R) | R927^d^ (S) | None (S) | C76^d^ (S) | None (S) | E21Q^g^,S95T^g^ (S,S) | None (S) | None (S) | L11^d^ (S) | Q360H^g^ (S) |
| 529 | None (R) | A908C (R) | E92D^g^,S100F^g^,A119D^g^,A193^d^,A205^d^(R) | R927^d^ (R) | None (R) | C76^d^ (R) | G406A^g^ (R) | E21Q^g^,D94G^f,g^,S95T^g^ (R,R) | V128G^f,g^ (R) | None (S) | L11^d^ (S) | Q360H^g^ (S) |
| 530 | None (R) | A908C (R) | E92D^g^,S100F^g^,A119D^g^,A193^d^,A205^d^(R) | R927^d^ (R) | C-12T (R) | C76^d^ (R) | G406A^g^ (R) | E21Q^g^,D94G^f,g^,S95T^g^ (R,R) | V128G^f,g^ (R) | None (S) | L11^d^ (S) | Q360H^g^ (S) |
| 531 | K88R^f,g^ (R) | None (R) | E92D^g^,S100F^g^,A205^d^ (R) | R927^d^ (R) | None (R) | C76^d^ (R) | M306I^f,g^,G175A^g^ (R) | E21Q^g^,S95T^g^ (R,R) | G78C^g^ (S) | None (R) | L11^d^ (R) | Q360H^g^ (S) |
| 533 | None (S) | None (S) | E92D^g^,S100F^g^,A205^d^ (S) | R927^d^ (R) | None (R) | C76^d^ (R) | A609^d,e^ (R) | E21Q^g^,S95T^g^ (R,R) | None (S) | None (S) | L11^d^ (S) | Q360H^g^ (R) |
| 539 | None (R) | None (R) | E92D^g^,S100F^g^,A205^d^ (R) | R927^d^ (R) | None (R) | C76^d^ (R) | None (R) | E21Q^g^,S95T^g^ (R,R) | None (R) | None (R) | L11^d^ (R) | Q360H^g^ (S) |
| 542 | None (S) | None (S) | E92D^g^,S100F^g^ (S) | R927^d^ (S) | None (S) | C76^d^ (S) | None (S) | E21Q^g^,S95T^g^ (S,S) | None (S) | None (S) | L11^d^ (S) | Q360H^g^ (S) |
| 550 | None (R) | None (R) | E92D^g^,S100F^g^,A205^d^ (R) | R927^d^ (R) | None (R) | C76^d^ (R) | None (R) | E21Q^g^,S95T^g^ (R,R) | None (R) | None (S) | L11^d^ (S) | Q360H^g^ (S) |
| 561 | None (S) | None (S) | E92D^g^,S100F^g^ (S) | R927^d^ (S) | None (S) | None (S) | None (S) | None (S,S) | None (S) | None (S) | None (S) | Q360H^g^ (S) |
| 587 | None (S) | None (S) | E92D^g^,S100F^g^,A205^d^ (S) | R927^d^ (S) | None (S) | C76^d^ (S) | None (S) | E21Q^g^,S95T^g^ (S,S) | None (S) | None (S) | L11^d^ (S) | Q360H^g^ (S) |
| 593 | None (S) | None (S) | E92D^g^,S100F^g^ (S) | R927^d^ (S) | None (S) | C76^d^ (S) | G406A^g^ (S) | E21Q^g^,S95T^g^ (R,R) | None (S) | None (S) | L11^d^ (S) | Q360H^g^ (R) |
| 596 | None (S) | None (S) | E92D^g^,S100F^g^ (S) | R927^d^ (S) | None (S) | C76^d^ (S) | None (S) | E21Q^g^,S95T^g^ (R,R) | None (S) | None (S) | L11^d^ (S) | Q360H^g^ (S) |
| 597 | None (S) | None (S) | E92D^g^,S100F^g^ (S) | R927^d^ (S) | None (S) | C76^d^ (S) | None (S) | E21Q^g^,S95T^g^ (R,R) | None (S) | None (S) | L11^d^ (S) | Q360H^g^ (S) |
| 606 | None (S) | None (S) | E92D^g^,S100F^g^ (S) | R927^d^ (S) | None (S) | C76^d^ (S) | None (S) | E21Q^g^,S95T^g^ (S,S) | None (S) | None (S) | L11^d^ (S) | Q360H^g^ (S) |
| 617 | None (S) | None (S) | E92D^g^,S100F^g^,A205^d^ (S) | R927^d^ (S) | None (S) | C76^d^ (S) | None (S) | None (S) | None (S) | None (S) | L11^d^ (S) | Q360H^g^ (S) |
| 672 | None (S) | None (S) | E92D^g^,S100F^g^ (S) | R927^d^ (S) | None (S) | C76^d^ (S) | None (S) | E21Q^g^,S95T^g^ (S,S) | None (S) | None (S) | L11^d^ (S) | Q360H^g^ (S) |
| 681 | None (S) | None (S) | E92D^g^,S100F^g^,A205^d^ (S) | R927^d^ (S) | None (S) | C76^d^ (S) | None (S) | None (S) | None (S) | None (S) | L11^d^ (S) | Q360H^g^ (S) |
| 693 | None (S) | None (S) | E92D^g^,S100F^g^ (S) | R927^d^ (S) | None (S) | None (S) | None (S) | E21Q^g^,S95T^g^ (S,S) | None (S) | None (S) | None (S) | Q360H^g^ (S) |
| 696 | K43R^f,g^ (R) | None (R) | E92D^g^,S100F^g^,A205^d^ (R) | R927^d^ (S) | None (S) | C76^d^ (S) | None (S) | E21Q^g^,S95T^g^ ( R,R) | None (S) | None (S) | L11^d^ (S) | Q360H^g^ (S) |
| 699 | None (S) | None (S) | E92D^g^,S100F^g^,A205^d^ (S) | R927^d^ (S) | None (S) | C76^d^ (S) | None (S) | E21Q^g^,S95T^g^ (S,S) | None (S) | None (S) | L11^d^ (S) | Q360H^g^ (S) |
| 720 | None (S) | None (S) | E92D^g^,S100F^g^,A205^d^ (S) | R927^d^ (S) | None (S) | C76^d^ (S) | None (S) | E21Q^g^,A90V^f,g^,S95T^g^ (R,R) | None (S) | None (S) | L11^d^ (S) | Q360H^g^ (S) |
| 764 | None (S) | None (S) | E92D^g^,S100F^g^,A205^d^ (S) | R927^d^ (S) | None (S) | C76^d^ (S) | None (S) | None (S) | None (S) | None (S) | L11^d^ (S) | Q360H^g^ (S) |
| 768 | None (S) | None (S) | E92D^g^,S100F^g^,A205^d^ (S) | R927^d^ (S) | None (S) | C76^d^ (S) | None (S) | None (S) | None (S) | None (S) | L11^d^ (S) | Q360H^g^ (S) |
| 770 | None (S) | None (S) | E92D^g^,S100F^g^ (S) | R927^d^ (S) | None (S) | C76^d^ (S) | None (S) | E21Q^g^,S95T^g^ (S,S) | None (S) | None (S) | L11^d^ (S) | Q360H^g^ (S) |
| 802 | None (S) | None (S) | E92D^g^,S100F^g^,A205^d^ (S) | R927^d^ (S) | None (S) | C76^d^ (S) | None (S) | E21Q^g^,S95T^g^ (S,S) | None (S) | None (S) | L11^d^ (S) | Q360H^g^ (S) |
| 804 | None (S) | None (S) | E92D^g^,S100F^g^,A205^d^ (S) | R927^d^ (S) | None (S) | C76^d^ (S) | None (S) | E21Q^g^,S95T^g^ (S,S) | None (S) | None (S) | L11^d^ (S) | Q360H^g^ (S) |
| 833 | None (S) | None (S) | E92D^g^,S100F^g^,A205^d^ (S) | R927^d^ (S) | None (S) | C76^d^ (S) | None (S) | E21Q^g^,S95T^g^ (S,S) | None (S) | None (S) | L11^d^ (S) | Q360H^g^ (S) |
| 835 | None (S) | None (S) | E92D^g^,S100F^g^,A205^d^ (S) | R927^d^ (S) | None (S) | C76^d^ (S) | None (S) | E21Q^g^,S95T^g^ (S,S) | None (S) | None (S) | L11^d^ (S) | Q360H^g^ (S) |
| 845 | None (S) | None (S) | E92D^g^,S100F^g^,A205^d^ (S) | R927^d^ (S) | None (S) | C76^d^ (S) | None (S) | E21Q^g^,S95T^g^ (S,S) | None (S) | None (S) | L11^d^ (S) | Q360H^g^ (S) |
| 853 | None (S) | None (S) | E92D^g^,S100F^g^,A205^d^ (S) | R927^d^ (S) | None (S) | C76^d^ (S) | None (S) | E21Q^g^,S95T^g^ (S,S) | None (S) | None (S) | L11^d^ (S) | Q360H^g^ (S) |
| 855 | None (S) | None (S) | E92D^g^,S100F^g^,A205^d^ (S) | R927^d^ (S) | None (S) | C76^d^ (S) | None (S) | E21Q^g^,S95T^g^ (S,S) | None (S) | None (S) | L11^d^ (S) | Q360H^g^ (S) |
| 876 | None (S) | None (S) | E92D^g^,S100F^g^,A205^d^ (S) | R927^d^ (S) | None (S) | C76^d^ (S) | None (S) | E21Q^g^,S95T^g^ (S,S) | None (S) | None (S) | L11^d^ (S) | Q360H^g^ (S) |
| 885 | None (S) | None (S) | E92D^g^,S100F^g^,A205^d^ (S) | R927^d^ (S) | None (S) | C76^d^ (S) | None (S) | E21Q^g^,S95T^g^ (S,S) | None (S) | None (S) | L11^d^ (S) | Q360H^g^ (S) |
| 912 | None (S) | None (S) | E92D^g^,S100F^g^,A205^d^ (S) | R927^d^ (S) | None (S) | C76^d^ (S) | None (S) | E21Q^g^,S95T^g^ (S,S) | None (S) | None (S) | L11^d^ (S) | Q360H^g^ (S) |
| 922 | K43R^f,g^ (R) | None (R) | E92D^g^,S100F^g^,A205^d^ (R) | R927^d^ (R) | None (R) | C76^d^ (R) | M306V^f,g^ (R) | E21Q^g^,S95T^g^ (S,S) | None (S) | None (S) | L11^d^ (S) | Q360H^g^ (S) |
| 928 | None (S) | None (S) | E92D^g^,S100F^g^,A205^d^ (S) | R927^d^ (S) | None (S) | C76^d^ (S) | N129S^e,g^ (S) | E21Q^g^,S95T^g^ (S,S) | None (S) | None (S) | L11^d^ (S) | Q360H^g^ (S) |
| 938 | K43R^f,g^ (R) | None (R) | E92D^g^,S100F^g^,A205^d^ (R) | R927^d^ (R) | None (R) | C76^d^ (R) | G406D^f,g^ (R) | E21Q^g^,D94Y^f,g^,S95T^g^ (R,R) | None (S) | None (S) | L11^d^ (S) | Q360H^g^ (R) |
| 967 | None (S) | None (S) | E92D^g^,S100F^g^,A205^d^ (S) | R927^d^ (S) | None (S) | C76^d^ (S) | None (S) | E21Q^g^,S95T^g^ (S,S) | None (S) | None (S) | L11^d^ (S) | Q360H^g^ (S) |
| 969 | K88R^f,g^ (R) | None (R) | E92D^g^,S100F^g^,A205^d^ (R) | R927^d^ (S) | None (S) | C76^d^ (S) | None (S) | E21Q^g^,S95T^g^ (S,S) | None (S) | None (S) | L11^d^ (S) | Q360H^g^ (S) |
| 970 | K43R^f,g^ (R) | None (R) | E92D^g^,S100F^g^,A205^d^ (R) | R927^d^ (S) | None (S) | C76^d^ (S) | None (S) | E21Q^g^,S95T^g^ (S,S) | None (S) | None (S) | L11^d^ (S) | Q360H^g^ (S) |
| 971 | None (S) | None (S) | E92D^g^,S100F^g^,A205^d^ (S) | R927^d^ (S) | None (S) | C76^d^ (S) | None (S) | E21Q^g^,S95T^g^ (S,S) | None (S) | None (S) | L11^d^ (S) | Q360H^g^ (S) |
| 972 | None (S) | None (S) | E92D^g^,S100F^g^,A205^d^ (S) | R927^d^ (S) | None (S) | C76^d^ (S) | None (S) | E21Q^g^,S95T^g^ (S,S) | None (S) | None (S) | L11^d^ (S) | Q360H^g^ (S) |
| 973 | None (S) | None (S) | E92D^g^,S100F^g^,A205^d^ (S) | R927^d^ (S) | None (S) | C76^d^ (S) | None (S) | E21Q^g^,S95T^g^ (S,S) | None (S) | None (S) | L11^d^ (S) | Q360H^g^ (S) |
| 975 | None (S) | None (S) | E92D^g^,S100F^g^,A205^d^ (S) | R927^d^ (S) | None (S) | C76^d^ (S) | None (S) | E21Q^g^,D94G^f,g^,S95T^g^ (R,R) | None (S) | None (S) | L11^d^ (S) | Q360H^g^ (S) |
| 976 | None (S) | None (S) | E92D^g^,S100F^g^,A205^d^ (S) | R927^d^ (S) | None (S) | C76^d^ (S) | None (S) | E21Q^g^,A90V^f,g^,S95T^g^ (R,R) | None (S) | None (S) | L11^d^ (S) | Q360H^g^ (S) |
| 978 | None (S) | None (S) | E92D^g^,S100F^g^,A205^d^ (S) | R927^d^ (S) | None (S) | C76^d^ (S) | None (S) | E21Q^g^,A90V^f,g^,S95T^g^ (R,R) | None (S) | None (S) | L11^d^ (S) | Q360H^g^ (S) |
| 980 | None (S) | None (S) | E92D^g^,S100F^g^,A205^d^ (S) | R927^d^ (S) | None (S) | C76^d^ (S) | None (S) | E21Q^g^,S95T^g^ (S,S) | None (S) | None (S) | L11^d^ (S) | Q360H^g^ (S) |
| 982 | None (S) | None (S) | E92D^g^,S100F^g^,A205^d^ (S) | R927^d^ (S) | None (S) | C76^d^ (S) | None (S) | E21Q^g^,S95T^g^ (S,S) | None (S) | None (S) | L11^d^ (S) | Q360H^g^ (S) |
| 985 | K43R^f,g^ (R) | None (R) | E92D^g^,S100F^g^,A205^d^ (R) | R927^d^ (R) | None (R) | C76^d^ (R) | G406S^f,g^ (R) | E21Q^g^,D94G^f,g^,S95T^g^ (R,R) | None (S) | A1401G(R) | L11^d^ (R) | Q360H^g^ (R) |
| 995 | None (S) | None (S) | E92D^g^,S100F^g^,A205^d^ (S) | R927^d^ (S) | None (S) | C76^d^ (S) | None (S) | E21Q^g^,S95T^g^ (S,S) | None (S) | None (S) | L11^d^ (S) | Q360H^g^ (S) |
| 996 | None (S) | None (S) | E92D^g^,S100F^g^,A205^d^ (S) | R927^d^ (S) | None (S) | C76^d^ (S) | None (S) | E21Q^g^,S95T^g^ (S,S) | None (S) | None (S) | L11^d^ (S) | Q360H^g^ (S) |
| 999 | None (S) | None (S) | E92D^g^,S100F^g^,A205^d^ (S) | R927^d^ (S) | None (S) | C76^d^ (S) | None (S) | E21Q^g^,S95T^g^ (S,S) | None (S) | None (S) | L11^d^ (S) | Q360H^g^ (S) |
| 1011 | None (S) | None (S) | E92D^g^,S100F^g^ (S) | R927^d^ (R) | None (R) | C76^d^ (R) | M306V^f,g^ (R) | E21Q^g^,S95T^g^ (S,S) | None (S) | None (S) | L11^d^ (S) | Q360H^g^ (S) |
| 1012 | None (S) | None (S) | E92D^g^,S100F^g^,A205^d^ (S) | R927^d^ (S) | None (S) | C76^d^ (S) | None (S) | None (S) | None (S) | None (R) | L11^d^ (R) | Q360H^g^ (S) |
| 1013 | K43R^f,g^ (R) | None (R) | E92D^g^,S100F^g^,A205^d^ (R) | R927^d^ (S) | None (S) | C76^d^ (S) | None (S) | E21Q^g^,S95T^g^ (S,S) | None (S) | None (S) | L11^d^ (S) | Q360H^g^ (S) |
| 1019 | None (S) | None (S) | E92D^g^,S100F^g^,A205^d^ (S) | R927^d^ (S) | None (S) | C76^d^ (S) | None (S) | E21Q^g^,S95T^g^ (S,S) | None (S) | None (S) | L11^d^ (S) | Q360H^g^ (S) |
| 1020 | None (S) | None (S) | E92D^g^,S100F^g^,A205^d^ (S) | R927^d^ (S) | None (S) | C76^d^ (S) | None (S) | E21Q^g^,S95T^g^ (S,S) | None (S) | None (S) | L11^d^ (S) | Q360H^g^ (S) |
| 1033 | None (R | None (R) | E92D^g^,S100F^g^,A205^d^ (R) | R927^d^ (R) | None (R) | C76^d^ (R) | M306V^f,g^ (R) | E21Q^g^,D94G^f,g^,S95T^g^ (R,R) | None (S) | None (S) | L11^d^ (S) | Q360H^g^ (S) |
| 1034 | None (S) | None (S) | E92D^g^,S100F^g^,A205^d^ (S) | R927^d^ (S) | None (S) | C76^d^ (S) | None (S) | E21Q^g^,S95T^g^ (S,S) | None (S) | None (S) | L11^d^ (S) | Q360H^g^ (S) |
| 1035 | K88R^f,g^ (R) | None (R) | E92D^g^,S100F^g^,A205^d^ (R) | R927^d^ (R) | None (R) | C76^d^ (R) | None (R) | E21Q^g^,S95T^g^ (S,S) | None (S) | None (S) | L11^d^ (S) | Q360H^g^ (S) |
| 1036 | None (S) | None (S) | E92D^g^,S100F^g^,A205^d^ (S) | R927^d^ (S) | None (S) | C76^d^ (S) | None (S) | E21Q^g^,S95T^g^ (S,S) | None (S) | None (S) | L11^d^ (S) | Q360H^g^ (S) |
| 1037 | None (S) | None (S) | E92D^g^,S100F^g^,A205^d^ (S) | R927^d^ (S) | None (S) | C76^d^ (S) | None (S) | E21Q^g^,S95T^g^ (S,S) | None (S) | None (S) | L11^d^ (S) | Q360H^g^ (S) |
| 1038 | None (S) | None (S) | E92D^g^,S100F^g^,A205^d^ (S) | R927^d^ (S) | None (S) | C76^d^ (S) | None (S) | E21Q^g^,S95T^g^ (S,S) | None (S) | None (S) | L11^d^ (S) | Q360H^g^ (S) |
| 1041 | None (S) | None (S) | E92D^g^,S100F^g^,A205^d^ (S) | R927^d^ (S) | None (S) | C76^d^ (S) | None (S) | E21Q^g^,S95T^g^ (S,S) | None (S) | None (S) | L11^d^ (S) | Q360H^g^ (S) |
| 1042 | None (S) | None (S) | E92D^g^,S100F^g^,A205^d^ (S) | R927^d^ (S) | None (S) | C76^d^ (S) | None (S) | E21Q^g^,S95T^g^ (S,S) | None (S) | None (S) | L11^d^ (S) | Q360H^g^ (S) |
| 1044 | None (S) | None (S) | E92D^g^,S100F^g^,A205^d^ (S) | R927^d^ (S) | None (S) | C76^d^ (S) | None (S) | E21Q^g^,S95T^g^ (S,S) | None (S) | None (S) | L11^d^ (S) | Q360H^g^ (S) |
| 1045 | None (S) | None (S) | E92D^g^,S100F^g^ (S) | R927^d^ (R) | None (R) | C76^d^ (R) | G314R^e,g^ (R) | E21Q^g^,S95T^g^ (S,S) | None (S) | None (S) | L11^d^ (S) | Q360H^g^ (S) |
| 1046 | None (S) | None (S) | E92D^g^,S100F^g^,A205^d^ (S) | R927^d^ (R) | None (R) | C76^d^ (R) | G2247(del)^e^ (R) | E21Q^g^,S95T^g^ (S,S) | None (S) | None (R) | L11^d^ (R) | Q360H^g^ (S) |
| 1048 | None (S) | None (S) | E92D^g^,S100F^g^,A205^d^ (S) | R927^d^ (S) | None (S) | C76^d^ (S) | None (S) | E21Q^g^,S95T^g^ (S,S) | None (S) | None (S) | L11^d^ (S) | Q360H^g^ (S) |
| 1050 | None (S) | None (S) | E92D^g^,S100F^g^,A205^d^ (S) | R927^d^ (R) | None (R) | C76^d^ (R) | None (R) | E21Q^g^,S95T^g^ (S,S) | None (S) | None (S) | L11^d^ (S) | Q360H^g^ (S) |
| 1051 | K43R^f,g^ (R) | None (R) | E92D^g^,S100F^g^,A205^d^ (S) | R927^d^ (R) | None (R) | C76^d^ (R) | None (R) | E21Q^g^,S95T^g^ (R,R) | None (S) | None (R) | L11^d^ (R) | Q360H^g^ (R) |
| 1052 | None (S) | None (S) | E92D^g^,S100F^g^,A205^d^ (S) | R927^d^ (R) | None (R) | C76^d^ (R) | None (R) | E21Q^g^,S95T^g^ (S,S) | None (S) | None (S) | L11^d^ (S) | Q360H^g^ (S) |
| 1053 | None (R) | None (R) | E92D^g^,S100F^g^,A205^d^ (S) | R927^d^ (R) | None (R) | C76^d^ (R) | None (R) | E21Q^g^,S95T^g^ (R,R) | None (R) | None (R) | L11^d^ (R) | Q360H^g^ (R) |
| 1054 | None (S) | None (S) | E92D^g^,S100F^g^,A205^d^ (S) | R927^d^ (S) | None (S) | C76^d^ (S) | None (S) | E21Q^g^,S95T^g^ (S,S) | None (S) | None (S) | L11^d^ (S) | Q360H^g^ (S) |
| 1055 | None (S) | None (S) | E92D^g^,S100F^g^,A205^d^ (S) | R927^d^ (S) | None (S) | C76^d^ (S) | None (S) | E21Q^g^,S95T^g^ (S,S) | None (S) | None (S) | L11^d^ (S) | Q360H^g^ (S) |
| 1056 | None (S) | None (S) | E92D^g^,S100F^g^,A205^d^ (S) | R927^d^ (S) | None (S) | C76^d^ (S) | None (S) | E21Q^g^,S95T^g^ (S,S) | None (S) | None (S) | L11^d^ (S) | Q360H^g^ (S) |
| 1057 | None (R) | None (R) | E92D^g^,S100F^g^,A205^d^ (S) | R927^d^ (R) | None (R) | C76^d^ (R) | None (R) | E21Q^g^,D94G^f,g^,S95T^g^ (R,R) | None (R) | None (S) | L11^d^ (R) | Q360H^g^ (R) |
| 1059 | None (R) | None (R) | E92D^g^,S100F^g^,A205^d^ (S) | R927^d^ (R) | None (R) | C76^d^ (R) | None (R) | E21Q^g^,S95T^g^ (R,R) | None (R) | None (S) | L11^d^ (R) | Q360H^g^ (R) |
| 1060 | None (S) | None (S) | E92D^g^,S100F^g^,A205^d^ (S) | R927^d^ (S) | None (S) | C76^d^ (S) | None (S) | E21Q^g^,S95T^g^ (S,S) | None (S) | None (S) | L11^d^ (S) | Q360H^g^ (S) |
| 1061 | None (S) | None (S) | E92D^g^,S100F^g^,A205^d^ (S) | R927^d^ (R) | None (R) | C76^d^ (R) | None (R) | E21Q^g^,S95T^g^ (S,S) | None (S) | None (S) | L11^d^ (S) | Q360H^g^ (R) |
| 1062 | None (S) | None (S) | E92D^g^,S100F^g^,A205^d^ (S) | R927^d^ (S) | None (S) | C76^d^ (S) | None (S) | E21Q^g^,S95T^g^ (S,S) | None (S) | None (S) | L11^d^ (S) | Q360H^g^ (S) |
| 1064 | None (S) | None (S) | E92D^g^,S100F^g^ (S) | R927^d^ (S) | None (S) | C76^d^ (S) | None (S) | E21Q^g^,S95T^g^ (S,S) | None (S) | None (S) | L11^d^ (S) | Q360H^g^ (S) |
| 1065 | None (R) | None (R) | E92D^g^,S100F^g^,A205^d^ (S) | R927^d^ (R) | None (R) | C76^d^ (R) | None (R) | E21Q^g^,S95T^g^ (R,R) | None (R) | None (S) | L11^d^ (R) | Q360H^g^ (R) |
| 1066 | None (S) | None (S) | E92D^g^,S100F^g^,A205^d^ (S) | R927^d^ (S) | None (S) | C76^d^ (S) | None (S) | E21Q^g^,S95T^g^ (S,S) | None (S) | None (S) | L11^d^ (S) | Q360H^g^ (S) |
| 1070 | None (S) | None (S) | E92D^g^,S100F^g^ (S) | R927^d^ (S) | None (S) | C76^d^ (S) | None (S) | E21Q^g^,S95T^g^ (S,S) | None (S) | None (S) | L11^d^ (S) | Q360H^g^ (S) |
| 1093 | None (S) | None (S) | E92D^g^,S100F^g^ (S) | R927^d^ (S) | None (S) | C76^d^ (S) | None (S) | E21Q^g^,S95T^g^ (S,S) | None (S) | None (S) | L11^d^ (S) | Q360H^g^ (S) |
| 1096 | None (S) | None (S) | E92D^g^,S100F^g^ (S) | R927^d^ (S) | None (S) | C76^d^ (S) | None (S) | E21Q^g^,S95T^g^ (R,R) | None (S) | None (S) | L11^d^ (S) | Q360H^g^ (S) |
| ^a^ “R”, resistance of isolates to the corresponding anti-TB drug; “S”, sensitivity of isolates to the corresponding anti-TB drug; “del”, deletion; “ins”, insertion; INH, isoniazid; RMP, rifampicin; SM, streptomycin; EMB, ethambutol; PZA, pyrazinamide; OFX, ofloxacin; LVX, levofloxacin; KAN, kanamycin; ETH, ethionamide; PAS, para-amino salicylic acid.  ^b^ intergenic regions.  ^c^ nucleotide mutational position is relative to *Mycobacterium tuberculosis* H37Rv *rpoB*, and amino acid position is relative to *Escherichia coli* numbering.  ^d^ synonymous.  ^e^ newly identified mutations.  ^f^ drug resistance-asssociated mutations with high confidence.  ^g^ non-synonymous. | | | | | | | | | | | | |
