# Supplementary material for: Molecular Epidemiology and Clinical Characteristics of Drug-Resistant Mycobacterium tuberculosis in a Tuberculosis Referral Hospital in China
Source: PLoS One. 2014 Oct 10;9(10):e110209. doi: 10.1371/journal.pone.0110209 (PMC4193878; doi:10.1371/journal.pone.0110209)
Supplement: Table S4 — Correlation between genotypic and phenotypic drug susceptibility testing results based on identification of reported drug resistance associated gene mutations. (DOC) [file pone.0110209.s004.doc]

| **Table S4.** The correlation between genotypic and phenotypic drug susceptibility testing results based on identification of reported drug resistance associated gene mutations. | | | |
| --- | --- | --- | --- |
| **Drugs^a^** | **No. of phenotypic resistant isolates** | **No. of genotypic resistant isolates** | **Percent (%)** |
| INH | 47 | 45 | 95.7 |
| RMP | 39 | 19 | 48.7 |
| SM | 37 | 17 | 45.9 |
| EMB | 34 | 16 | 47.1 |
| PZA | 15 | 6 | 40 |
| OLX, LVX | 41 | 17 | 41.5 |
| KAN | 20 | 4 | 20 |
| ETH | 17 | 0 | 0 |
| PAS | 24 | 0 | 0 |
| ^a^ INH, isoniazid; RMP, rifampicin; SM, streptomycin; EMB, ethambutol; PZA, pyrazinamide; OFX, ofloxacin; LVX, levofloxacin; KAN, kanamycin; ETH, ethionamide; PAS, para-amino salicylic acid. | | | |
