# Supplementary material for: Molecular Epidemiology and Clinical Characteristics of Drug-Resistant Mycobacterium tuberculosis in a Tuberculosis Referral Hospital in China
Source: PLoS One. 2014 Oct 10;9(10):e110209. doi: 10.1371/journal.pone.0110209 (PMC4193878; doi:10.1371/journal.pone.0110209)
Supplement: Table S5 — Drug resistance profiles and 28 loci MIRU-VNTR results of M. tuberculosis isolates. (DOC) [file pone.0110209.s005.doc]

| **Table S5.** Drug resistance profiles and 28 loci MIRU-VNTR results of *M. tuberculosis* isolates. | | | | |
| --- | --- | --- | --- | --- |
| **Isolates** | **Drug resistance^a^** | **Type of isolates** | **VNTR-28 loci** | **Beijing/W genotypes** |
| 26 | None | Susceptible | 233324163533-4443345824328b38 | Beijing |
| 39 | None | Susceptible | 233324163533-4543445824328b3e | Beijing |
| 143 | INH, RMP, SM, EMB, PZA,OFX, LVX, KAN, PAS, ETH | XDR | 232224153433-4543445824327d3e | Beijing |
| 119 | INH,OFX,LVX,KAN | Other | 232324163533-4543446824328b8f | Beijing |
| 130 | SM | Other | 231324163533-244344783432ab7c | Beijing |
| 180 | INH,RMP, EMB,PAS | MDR | 233324163533-3543445824328dac | Beijing |
| 185 | INH, RMP,SM,EMB,PZA,OFX,LVX,KAN,PAS,ETH | XDR | 233324163533-4543446822328b3d | Beijing |
| 193 | INH, RMP, SM, EMB, OFX, LVX | MDR | 213224163433-2433445724225d3c | Beijing |
| 195 | INH, RMP, SM,OFX, LVX, KAN | XDR | 233224163433-2543446824328b3e | Beijing |
| 196 | INH, RMP, SM,OFX, LVX, KAN, PAS | XDR | 233224163433-4643442824328d3e | Beijing |
| 201 | INH, RMP, SM, EMB,OFX, LVX, KAN, PAS | XDR | 233224153433-454344482432ab8c | Beijing |
| 206 | INH,SM,ETH | Other | 234324163533-4333344934328b8d | Beijing |
| 209 | INH,SM,EMB,OFX,LVX | Other | 232324163333-4443444824320b3e | Beijing |
| 216 | INH | Other | 252324142322-4121424424328b33 | non-Beijing |
| 218 | INH,SM,PZA,OFX,LVX,PAS | Other | 233324163543-4543446834322dac | Beijing |
| 221 | INH,RMP,SM,EMB,OFX,LVX,KAN,PAS,ETH | XDR | 233324173533-4543445824320dac | Beijing |
| 227 | None | Susceptible | 233325163533-4543446824328dae | Beijing |
| 248 | INH,RMP,SM,EMB,PZA,OFX,LVX,KAN,PAS,ETH | XDR | 233324163533-4543445824328b28 | Beijing |
| 262 | INH, RMP, SM, EMB, PZA,OFX, LVX, PAS | MDR | 233224163433-4543444824328dae | Beijing |
| 292 | INH,RMP, OFX,LVX,PAS,ETH | MDR | 233324163523-4543446824329dae | Beijing |
| 317 | SM,ETH | Other | 233324153533-4543446d24328bae | Beijing |
| 316 | INH, RMP, SM | MDR | 233224163433-4443445844328bae | Beijing |
| 322 | INH,RMP,EMB,PZA,OLX,LVX,KAN,PAS | XDR | 222324153533-3543446824328dae | Beijing |
| 471 | EMB,PZA, OFX,LVX, PAS | Other | 233324163533-4543446824328dac | Beijing |
| 477 | INH, RMP, SM, PZA, OFX, LVX | MDR | 233223153433-1443444734328bac | Beijing |
| 489 | None | Susceptible | 233324163433-4543354424328cae | Beijing |
| 517 | INH,PZA,OFX,LVX,PAS,ETH | Other | 233324163533-4543446822328a8e | Beijing |
| 523 | INH, RMP, SM, EMB, PZA,OFX, LVX, KAN | XDR | 233224153433-4543444824328bac | Beijing |
| 525 | INH,RMP,SM | MDR | 233224163533-4543446724328bae | Beijing |
| 529 | INH, RMP, SM, EMB, PZA, OFX, LVX | MDR | 233223153433-1443444734328bae | Beijing |
| 530 | INH, RMP, SM, EMB, PZA, OFX, LVX | MDR | 233223153433-1443444734327bab | Beijing |
| 531 | INH, RMP, SM, EMB,OFX, LVX, KAN | XDR | 233223153434-4543445824328bae | Beijing |
| 533 | EMB,OFX,LVX,PAS, ETH | Other | 233324163533-4543446d24328eaf | Beijing |
| 539 | INH,RMP,SM,EMB,PZA,PAS,OFX,LVX,KAN | XDR | 233224164533-4543445724328aae | Beijing |
| 542 | INH | Other | 236224113322-3443442634329a36 | non-Beijing |
| 550 | INH,RMP,SM,EMB,PZA, PAS,OFX,LVX | MDR | 233224173533-4543445824328bac | Beijing |
| 561 | None | Susceptible | 223224163533-4543346824318bae | Beijing |
| 587 | None | Susceptible | 233224163533-4543445824328bac | Beijing |
| 593 | INH,RMP,OFX,LVX,PAS,ETH | MDR | 233324173533-3543445824328a3c | Beijing |
| 596 | INH,RMP,OFX,LVX, PAS | MDR | 2a3224133330-4543445824328d3e | Beijing |
| 597 | INH, RMP,OFX,LVX,PAS | MDR | 2a3224133330-3543445824328d3e | Beijing |
| 606 | None | Susceptible | 233224142323-2323346724338d44 | Beijing |
| 617 | None | Susceptible | 233324163531-4744446824328dac | Beijing |
| 672 | None | Susceptible | 233225123323-2225345524315d12 | Beijing |
| 681 | None | Susceptible | 233324163533-4543443924328dae | Beijing |
| 693 | None | Susceptible | 233324163533-4544435824328d3c | Beijing |
| 696 | INH,RMP,SM,OFX,LVX | MDR | 233324163533-4543446824225dae | Beijing |
| 699 | None | Susceptible | 233324163533-4543444724328dac | Beijing |
| 720 | RMP,OFX,LVX | Other | 233324163533-4543446824325daa | Beijing |
| 764 | None | Susceptible | 233325153533-4447445834328da4 | Beijing |
| 768 | None | Susceptible | 232224163423-454344482432adaa | Beijing |
| 770 | None | Susceptible | 232224143423-3343445824328daa | Beijing |
| 802 | None | Susceptible | 233324143543-444344772432bdac | Beijing |
| 804 | None | Susceptible | 233324163533-4523345824328d8e | Beijing |
| 833 | None | Susceptible | 233224163533-4543445524328b7e | Beijing |
| 835 | None | Susceptible | 232225133533-4543444824328b1e | Beijing |
| 845 | None | Susceptible | 232224163533-4543446824328b4e | Beijing |
| 853 | None | Susceptible | 232324163533-4543445724328a4e | Beijing |
| 855 | None | Susceptible | 232324163533-2423353624315d86 | Beijing |
| 876 | None | Susceptible | 233324163533-454444682432853e | Beijing |
| 885 | None | Susceptible | 233324163533-4544346824325b3f | Beijing |
| 912 | None | Susceptible | 233324163533-4544345824328bae | Beijing |
| 922 | INH,RMP,SM,EMB | MDR | 233324163533-4544346824326d3e | Beijing |
| 928 | None | Susceptible | 233324153531-4343445924328dae | Beijing |
| 938 | INH,RMP,SM, EMB,PAS,OFX,LVX,ETH | MDR | 233324143533-2543446724328dac | Beijing |
| 967 | None | Susceptible | 233224163533-4543446824328bae | Beijing |
| 969 | SM | Other | 233324163533-4543426724327baf | Beijing |
| 970 | INH,SM | Other | 233324173533-4533344824328dae | Beijing |
| 971 | None | Susceptible | 233224163133-4543446824328bae | Beijing |
| 972 | None | Susceptible | 233224163533-4543444824328bac | Beijing |
| 973 | INH,RMP | MDR | 233224163533-4543446824328bae | Beijing |
| 975 | OFX,LVX | Other | 253324153533-4533446824328bae | Beijing |
| 976 | OFX,LVX | Other | 233424163533-2643446824322baf | Beijing |
| 978 | OFX,LVX | Other | 233324163533-2843446824322daf | Beijing |
| 980 | None | Susceptible | 233224163533-4643345824327bad | Beijing |
| 982 | None | Susceptible | 233224153533-4543446824328bae | Beijing |
| 985 | INH,RMP,SM,EMB,OFX,LVX,KAN | XDR | 233424173534-2543444824328bad | Beijing |
| 995 | None | Susceptible | 233224163534-4543445824328dae | Beijing |
| 996 | None | Susceptible | 232223153533-4543446724328ba7 | Beijing |
| 999 | None | Susceptible | 233224153533-3344446634317ba8 | Beijing |
| 1011 | EMB | Other | 232124154313-2353344824325b45 | Beijing |
| 1012 | KAN | Other | 233224161523-445344784432ad5c | non-Beijing |
| 1013 | INH,RMP,SM | MDR | 233224153523-4543445824328bae | Beijing |
| 1019 | None | Susceptible | 233224153523-4543446824428bae | Beijing |
| 1020 | None | Susceptible | 233224163533-4543446824325bae | Beijing |
| 1033 | INH,RMP,SM,EMB,OFX,LVX | MDR | 233124163523-2433445724326bae | Beijing |
| 1034 | None | Susceptible | 213224163523-2433445724226dae | Beijing |
| 1035 | INH,SM,EMB | Other | 213224163523-2543445724326dae | Beijing |
| 1036 | None | Susceptible | 213224163523-2433445724226dae | Beijing |
| 1037 | None | Susceptible | 213224163523-2433445724226dae | Beijing |
| 1038 | None | Susceptible | 213224163523-2433445724226dae | Beijing |
| 1041 | None | Susceptible | 213224163523-2433445724226bae | Beijing |
| 1042 | None | Susceptible | 213224163523-2433445724226b9e | Beijing |
| 1044 | None | Susceptible | 213224163523-2433445724226b9e | non-Beijing |
| 1045 | EMB | Other | 212224153325-2323344824315b94 | Beijing |
| 1046 | EMB,KAN | Other | 233224163533-2433445724226b2e | Beijing |
| 1048 | None | Susceptible | 213224163533-4443446734326b2e | Beijing |
| 1050 | EMB | Other | 213224163533-2433445724226bae | Beijing |
| 1051 | INH,RMP,SM,EMB,OLX,LVX,KAN,PAS,ETH | XDR | 213224163533-4543445824326dae | Beijing |
| 1052 | EMB | Other | 213224163533-2433445724226dae | Beijing |
| 1053 | INH,RMP,SM,EMB,PZA,OFX,LVX,KAN,PAS,ETH | XDR | 213224163533-2433445724226da5 | Beijing |
| 1054 | None | Susceptible | 213224163533-2433445724226da5 | Beijing |
| 1055 | None | Susceptible | 213224163533-2433445724226da5 | Beijing |
| 1056 | None | Susceptible | 213224163533-2433445724226dae | Beijing |
| 1057 | INH,RMP,SM,EMB,OLX,LVX,KAN,PAS,ETH | XDR | 213224163533-2433445724226da5 | Beijing |
| 1059 | INH,RMP,SM,EMB,OLX,LVX,KAN,PAS,ETH | XDR | 213224163533-2433445724226dae | Beijing |
| 1060 | None | Susceptible | 212224163633-3333445834326dac | Beijing |
| 1061 | INH,RMP,EMB,ETH | MDR | 213224163533-2433445724226dac | Beijing |
| 1062 | None | Susceptible | 213224163533-2433445724226bae | Beijing |
| 1064 | None | Susceptible | 213224163533-2433445724226bae | Beijing |
| 1065 | INH,RMP,SM,EMB,PAS,OLX, LVX ,KAN,ETH | XDR | 213224163533-2433445724226dae | Beijing |
| 1066 | None | Susceptible | 213224163533-2433445724226dae | Beijing |
| 1070 | None | Susceptible | 213124163533-4543436824326dae | Beijing |
| 1093 | None | Susceptible | 233224153533-4443444624326dac | Beijing |
| 1096 | INH,RMP,OLX, LVX | MDR | 233224163433-4443445724326a3e | Beijing |
| ^a^ INH, isoniazid; RMP, rifampicin; SM, streptomycin; EMB, ethambutol; PZA, pyrazinamide; OFX, ofloxacin; LVX, levofloxacin; KAN, kanamycin; ETH, ethionamide; PAS, para-amino salicylic acid. | | | | |
