# Supplementary material for: Molecular Epidemiology and Clinical Characteristics of Drug-Resistant Mycobacterium tuberculosis in a Tuberculosis Referral Hospital in China
Source: PLoS One. 2014 Oct 10;9(10):e110209. doi: 10.1371/journal.pone.0110209 (PMC4193878; doi:10.1371/journal.pone.0110209)
Supplement: Table S6 — MIRU-VNTR fingerprinting results for 20 clustered Mycobacterium tuberculosis isolates. (DOC) [file pone.0110209.s006.doc]

| **Table S6.** MIRU-VNTR fingerprinting results for 20 clustered *Mycobacterium tuberculosis* isolates. | |
| --- | --- |
| **MIRU pattern** | **Frequency** |
| 213224163533-2433445724226dae | 5 (Isolate No. 1052,1056, 1059, 1065, 1066) |
| 213224163533-2433445724226da5 | 4 (Isolate No.1053,1054,1055,1057) |
| 213224163523-2433445724226dae | 4 (Isolate No. 1034,1036,1037,1038) |
| 213224163533-2433445724226bae | 3 (Isolate No.1050,1062,1064) |
| 233224163533-4543446824328bae | 2 (Isolate No. 967, 973) |
| 213224163523-2433445724226b9e | 2 (Isolate No. 1042, 1044) |
|  | |
