# Supplementary material for: Molecular Epidemiology and Clinical Characteristics of Drug-Resistant Mycobacterium tuberculosis in a Tuberculosis Referral Hospital in China
Source: PLoS One. 2014 Oct 10;9(10):e110209. doi: 10.1371/journal.pone.0110209 (PMC4193878; doi:10.1371/journal.pone.0110209)
Supplement: Table S7 — Drug resistance profiles and epidemiological information of the clustered isolates. (DOC) [file pone.0110209.s007.doc]

| **Table S7.** Drug resistance profiles and epidemiological information of the clustered isolates. | | | | | | | | | | | | | |
| --- | --- | --- | --- | --- | --- | --- | --- | --- | --- | --- | --- | --- | --- |
| **Isolates** | **Type of isolates** | | **Phenotypic drug resistance profiles^a^** | **Genotypic drug resistance profiles** | **Age** | **Gender** | **Geographic**  **location** | **Treatment**  **history** | **Hospital location** | **Date of hospitalization** | **Underlying diseases** | **Diagnosis** | **Clinical outcomes** |
| **2132241635332433445724226dae** | | | | | | | | | | | | | |
| 1052 | Other | | EMB | None | 50 | Male | Beijing | Retreated | Tuberculosis ward 1 | 2010.11.24-2010.12.1 | Diabetes mellitus, fatty liver, abnormal liver function | Secondary pulmonary tuberculosis | Alive |
| 1056 | susceptible | | None | None | 45 | Male | Beijing | New | Tuberculosis ward 2 | 2010.11.6-2010.11.26 | Diabetes mellitus, Pulmonary infection, abnormal liver function, coronary heart disease | Secondary pulmonary tuberculosis | Alive |
| 1059 | XDR | | INH,RMP,SM,EMB,OLX,LVX,KAN,PAS,ETH | INH:*katG*(R463L),*accD6*(D229G) | 50 | Female | Anhui | New | Tuberculosis ward 2 | 2010.11.18-2011.1.17 | Pulmonary infection, abnormal liver function | Secondary pulmonary tuberculosis, tuberculous pleurisy | Alive |
| 1065 | XDR | | INH,RMP,SM,EMB,PAS,OLX, LVX ,KAN,ETH | INH:*katG*(R463L),*accD6*(D229G) | 41 | Female | Henan | Retreated | Tuberculosis ward 1 | 2009.6.20-2009.6.23 | None | Secondary pulmonary tuberculosis | Alive |
| 1066 | susceptible | | None | None | 17 | Female | Anhui | Retreated | Tuberculosis ward 1 | 2011.11.28-2011.11.30 | Hyperuricemia | Secondary pulmonary tuberculosis, bronchial tuberculosis, tuberculous pleurisy | Alive |
| **213224163533-2433445724226da5** | | | | | | | | | | | | | |
| 1053 | XDR | | INH,RMP,SM,EMB,PZA,OFX,LVX,KAN,PAS,ETH | INH:*katG*(R463L),*accD6*(D229G) | 19 | Female | Hebei | New | Tuberculosis ward 3 | 2010.11.1-2010.11.24 | None | Pulmonary tuberculosis, bronchial tuberculosis | Alive |
| 1054 | susceptible | | None | None | 34 | Male | Henan | Retreated | Tuberculosis ward 3 | 2010.1.11-2010.1.21 | Pulmonary infection | Secondary pulmonary tuberculosis, tuberculous pleurisy | Alive |
| 1055 | susceptible | | None | None | 81 | Female | Heilongjiang | New | Tuberculosis ward ICU | 2010.11.1-2010.11.18 | Pulmonary infection, Chronic bronchitis | Secondary pulmonary tuberculosis, bronchial tuberculosis, tuberculous pleurisy, respiratory failure | Died |
| 1057 | XDR | | INH,RMP,SM,EMB,OLX,LVX,KAN,PAS,ETH | INH:*katG*(R463L),*accD6*(D229G);OLX,LVX:*gyrA*(D94G) | 34 | Female | Shanxi | Retreated | Tuberculosis ward 3 | 2010.11.5-2010.12.24 | None | Secondary pulmonary tuberculosis | Alive |
| **213224163523-2433445724226dae** | | | | | | | | | | | | | |
| 1034 | | susceptible | None | None | 27 | Male | Hebei | Retreated | Tuberculosis ward 1 | 2010.3.10-2010.5.7 | None | Secondary pulmonary tuberculosis | Alive |
| 1036 | | susceptible | None | None | 27 | Female | Shanxi | Retreated | Tuberculosis ward 1 | 2010.9.20-2010.10.29 | Pulmonary infection | Secondary pulmonary tuberculosis, bronchial tuberculosis | Alive |
| 1037 | | susceptible | None | None | 35 | Male | Jilin | Retreated | Tuberculosis ward 2 | 2009.7.9-2009.9.23 | Pulmonary infection | Secondary pulmonary tuberculosis | Alive |
| 1038 | | susceptible | None | None | 76 | Female | Niaoning | New | Orthopaedic ward 1 | 2010.9.28-2010.11.13 | Diabetes mellitus, [hypertension](javascript:void(0);) | Secondary pulmonary tuberculosis, bone tuberculosis | Alive |
| **213224163533-2433445724226bae** | | | | | | | | | | | | | |
| 1050 | | Other | EMB | None | 47 | Female | Hubei | New | Tuberculosis ward 2 | 2010.10.28-2010.11.5 | None | Secondary pulmonary tuberculosis, bronchial tuberculosis | Alive |
| 1062 | | susceptible | None | None | 19 | Female | Shanxi | Retreated | Tuberculosis ward 3 | 2010.11.30-2011.1.14 | Pulmonary infection | Secondary pulmonary tuberculosis, bronchial tuberculosis, mediastinal tuberculous lymphadenitis | Alive |
| 1064 | | susceptible | None | None | 74 | Male | Heilongjiang | Retreated | Tuberculosis ward 2 | 2010.11.29-2011.1.30 | Pulmonary infection | Secondary pulmonary tuberculosis, tuberculous pleurisy | Alive |
| **233224163533-4543446824328bae** | | | | | | | | | | | | | |
| 967 | | susceptible | None | None | 73 | Male | Beijing | Retreated | Tuberculosis ward 3 | 2009.9.16-2009.10.8 | Abnormal liver function | Secondary pulmonary tuberculosis | Alive |
| 973 | | MDR | INH,RMP | INH:*katG*(R463L),*accD6*(D229G);RMP:*rpoB*(D516A,H526R) | 39 | Male | Beijing | Retreated | Tuberculosis ward 1 | 2010.5.21-2010.6.28 | Diabetes mellitus, fatty liver | Secondary pulmonary tuberculosis, tuberculous pleuritis | Alive |
| **213224163523-2433445724226b9e** | | | | | | | | | | | | | |
| 1042 | | susceptible | None | None | 24 | Female | Beijing | Retreated | Tuberculosis ward 4 | 2010.3.3-2010.5.19 | Pulmonary infection, abnormal liver function | Secondary pulmonary tuberculosis | Alive |
| 1044 | | susceptible | None | None | 73 | Female | Hebei | Retreated | Tuberculosis ward 1 | 2010.6.9-2010.7.26 | Hypertention,bronchitis,chronic lung disease, heart disease, parkinsonism | Secondary pulmonary tuberculosis, | Alive |
| ^a^ INH, isoniazid; RMP, rifampicin; SM, streptomycin; EMB, ethambutol; PZA, pyrazinamide; OFX, ofloxacin; LVX, levofloxacin; KAN, kanamycin; ETH, ethionamide; PAS, para-amino salicylic acid. | | | | | | | | | | | | | |
